# Towards simulation optimization of subway station considering refined passenger behaviors

**DOI:** 10.1371/journal.pone.0304081

**Published:** 2024-06-06

**Authors:** Yingping Wang, Rui Yuan, Xueying Tong, Zongning Bai, Yutong Hou

**Affiliations:** 1 Transport planning and Research Institute, Ministry of Transport, Beijing, 100028, China; 2 Beijing University of Technology, Beijing, 100124, China; Universidad de Sevilla Facultad de Ciencias Económicas y Empresariales: Universidad de Sevilla Facultad de Ciencias Economicas y Empresariales, SPAIN

## Abstract

The escalating passenger flow in subway systems presents significant challenges to station facilities during peak hours. Poorly designed station facilities can reduce passenger throughput efficiency and compromise passenger safety. This study conducts on-site investigations to extract refined parameters of passenger behaviors in security check and ticket checking areas. Using Beijing Subway Yizhuang Line Ciqunan Station as a case study, a microscopic simulation model is developed to replicate pedestrian flow within the subway station. By focusing on passenger demand and traffic organization, the layout of station facilities is regulated and optimized. After optimization, the passenger density in the security check and ticket inspection areas during the morning peak period decreased from 1.33 people/m^2^ to 1.00 people/m^2^; the longest queue length on the east side decreased from 15 people to 10 people, and the maximum queue length on the west side decreased from 7 people to 3 people. During peak hours, the dispersal time of passenger flow on the west side when entering the station decreased from 31.56 minutes to 30.04 minutes, and on the east side, it decreased from 36.12 minutes to 30.87 minutes. The optimization results effectively improved the efficiency of entering the station during peak hours.

## 1 Introduction

Rail transit, as a cornerstone of urban public transportation, offers advantages such as large capacity, high speed, and environmental friendliness. With the growing passenger flow, congestion frequency has also increased. The Ministry of Transport has issued the "Management Measures for the Evaluation and Management of Urban Rail Transit Service Quality," which explicitly emphasizes the importance of providing high-quality services to various types of passengers. To address congestion and enhance passenger service quality, current research focuses on targeted optimization of station facilities considering refined passenger behaviors during the morning peak period. This period is characterized by concentrated entry periods, large flow volumes, and clear behavioral purposes, primarily commuting passengers, leading to congestion and long waiting times. In response, it is essential to consider the refined needs of passengers and improve security check and ticket checking facilities to provide efficient passage services for different types of passengers.

Compared to macroscopic control measures that consider overall passenger flow data, pedestrian traffic simulation software offers a new approach for quantitatively evaluating the refined characteristics of passenger micro-travel behaviors within rail transit stations. It can also assess the rationality and efficiency of service facilities within rail transit stations under design or optimization. Pedestrian flow simulation software provides data such as heat density maps, maximum queue lengths, and system dissipation times within stations, enabling quantitative analysis and evaluation of service facilities. However, existing simulation software often inadequately considers passengers’ micro-travel behaviors and lacks a comprehensive evaluation system for service facility parameters, failing to meet the diverse needs for analyzing passenger behavior characteristics. Therefore, integrating passengers’ micro-scale behaviors into the macroscopic facility evaluation system for subway stations is essential for refined passenger behavior traffic simulation analysis.

Regarding passengers’ behavior at key facilities within subway stations, scholars have primarily focused on service time and efficiency. For instance, Li and Guo [1] proposed a pedestrian boarding and alighting strategy to alleviate the congestion of bidirectional flow bottleneck caused by the boarding and alighting process on the platform. Yang et al. [2] proposed to alleviate congestion in key areas of subway stations by adding guidance, and constructed the correlation between guidance and congestion through a multi-objective optimization model to provide the optimal guidance path. Wu et al. [3] found that passenger flow line can be changed through railings, which can balance the utilization rate of each exit and relieve the congestion of exit bottlenecks. Shi et al. [4] conducted a decongestion solution for the bottleneck of the turning channel of subway station, that was, to increase the width of the intersection. Cheng et al. [5] and Sun et al. [6] believed that elevators and staircases are the most congestion area of subway station, which can relieve by setting guardrails. Xu et al. [7] concluded that escalators and stairs was critical points for decongestion, while the removal of metal barriers was a useful decongestion solution. Wu et al. [8] proposed the decongestion solutions that installing additional railings to disperse passenger flow and adding more ticket gates. Soltani et al. [9] used two software programs, Aimsun and Path Finder, to examine the service levels of pedestrian pathways and corridors at the Sadeghiyeh urban train station in Tehran, indicated the service levels of the main hall, north entrance, and south corridor of the station. Additionally, numerous scholars have made multiple achievements in areas such as simulating pedestrian behavior in subway stations [10,11], airport terminals [12], stairs [13], irregular channels (L [14], T [15], Y [16], Z [17]), and evacuation conditions [11,18,19].

The arrangement of facilities within subway stations significantly influences passengers’ path choices. Wang et al. [20] focused on fare-paying passengers entering the station, analyzing the behavior of entering passengers, the selection of facilities within the fare-paying area, and evaluating the safety and reliability of the subway station’s fare-paying area based on three indicators: service level, queue length, and space occupancy. Chen et al. [21] analyzed the movement characteristics of heterogeneous pedestrians in the ticket-checking facility area based on gender, age, luggage-carrying status, and accompanying status, and derived solutions for optimizing the layout of ticket-checking facilities at the entrance. Antonova et al. [22] used Anylogic software for simulation and parameter adjustment to optimize the existing ticketing and checking systems at subway stations. Azadpeyma et al. [23] used the VISSIM software to simulate passenger flow at the Shohada Square subway station under six different scenarios. The results indicated that the decision to remove manned ticketing positions would improve the performance of the controlled airspace section by 43%. Huang et al. [24] proposed evaluation indices for pedestrian flow intersection areas, pedestrian conflict points, local congestion indices, and facility utilization inequality coefficients, building a simulation model of passenger flow in the subway station hall based on the Anylogic pedestrian library simulation platform.

Current pedestrian flow research primarily focuses on establishing models that accurately represent passengers’ actual travel paths. Previous studies have provided pedestrian flow models such as social force models or cellular automaton models, while further research can be conducted on the specific micro-behaviors of passengers. The refinement of passenger behavior research compared to traditional macro models can more accurately describe the characteristics exhibited by different passenger groups, revealing the travel characteristics of passenger groups from a micro-individual perspective, providing accuracy for simulation results, and offering strategic guidance for the planning and design of rail transit stations.

This paper simulates the refinement of passenger behavior using Beijing’s subway system as an example. Based on field surveys, the heterogeneity of passenger flow through subway station facilities and influencing factors are analyzed. Anylogic simulation software is employed to calibrate heterogeneous passenger behavior. Using Beijing Subway’s Yizhuang Line Ciqunan Station as an example, a microsimulation model is established to simulate the travel process of pedestrian flow within the subway station, providing station facility layout regulation and optimization from the perspectives of passenger demand and crowd organization. Finally, the optimization plan is validated through comparisons of area density maps, maximum queue lengths, and system dissipation times. This paper is of significant importance for optimizing subway station facility layouts and improving subway service quality.

## 2 Passenger behavior and influencing factors

### 2.1 Data survey and processing

The Code for Design of Metro [25] specifies the maximum capacity of various parts in the station. The maximum passage capacity of ticket checking machines is much smaller than that of other parts. Many scholars have also studied the bottleneck areas within stations, and the results indicate that security check facilities and ticket checking facilities are the main bottleneck locations of subway stations, constraining the operational efficiency of the station. Therefore, this paper selects security checks and ticket checking as the research objects. Through methods such as field investigations and video analysis, it analyzes the time taken by different types of passengers to pass through these two types of facilities and studies their main influencing factors.

The study randomly selected 10 stations from 5 subway lines in Beijing for investigation, from March 13th (Monday) to March 17th (Friday) in 2023, during the morning peak hours from 7:00 to 8:00, the selected stations are shown in Table 1. Nota that, the COVID-19 pandemic and the Spring Festival holiday had ended in this period, and the passenger flow on the Beijing subway had returned to normal levels. The data collected from on-site surveys and video analysis were normalized. A total of 1341 sets of passenger data were collected, including 528 sets in the security check area and 813 sets in the ticket checking area, comprising 717 male passengers and 624 female passengers.

**Table 1:** Selected stations.

Line	Station	Line	Station
Line 5	Tiantongyuanbei	Line 14	Pingleyuan
Line 5	Tiantongyuan	Line 17	Beishenshu
Line 7	Jiulongshan	Line 17	Ciqubei
Line 7	Baiziwan	Yizhuang Line	Xiaohongmen
Line 14	Beigongdaximen	Yizhuang Line	Ciqunan

**Fig 1:**
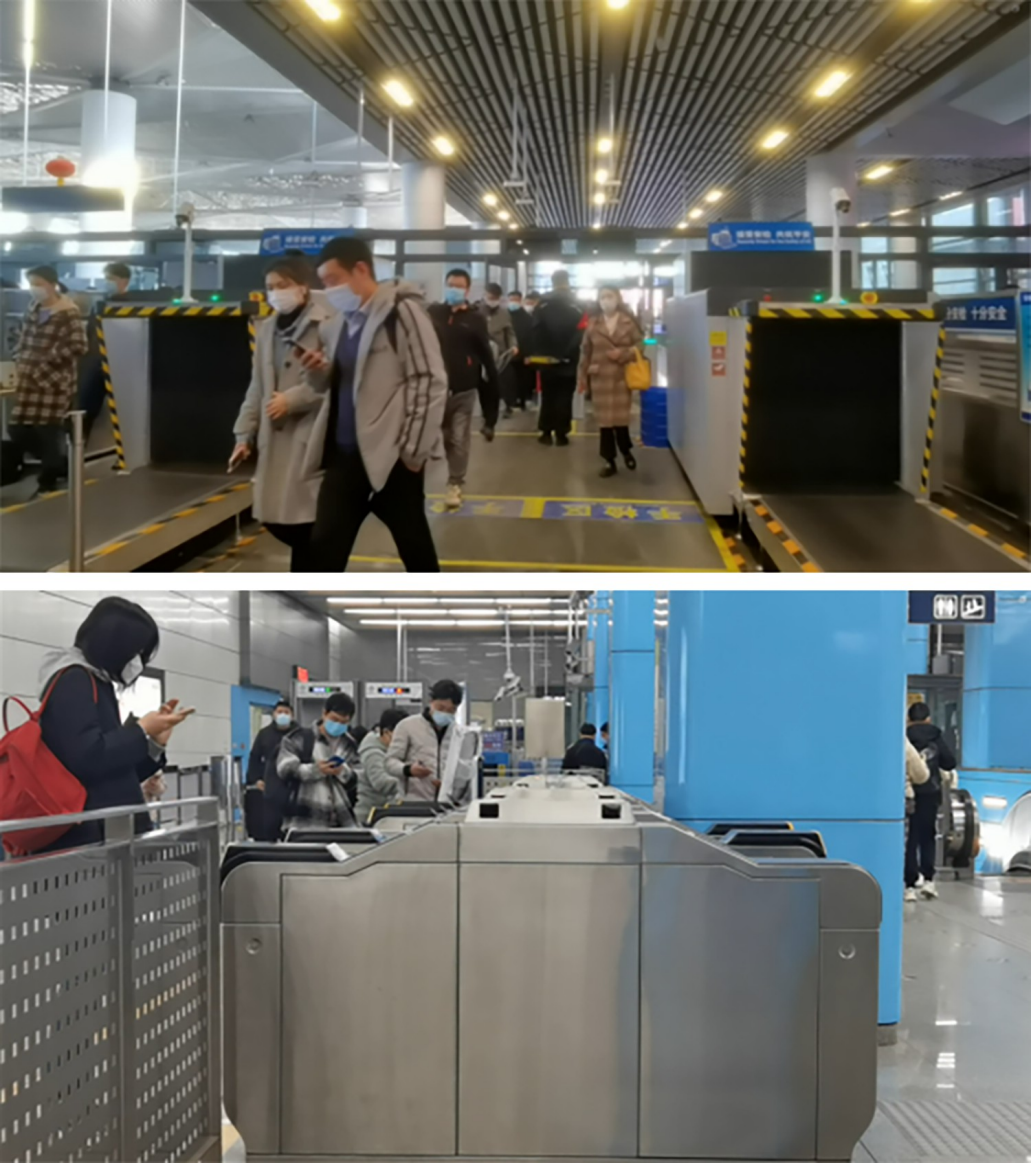
(a) Field investigation area. (b) Field investigation area.

Specifically, we set up cameras at the security check and ticketing areas respectively (as shown in Fig 1), and collected the average passenger flow per unit time passing through the two areas, the attributes of all passengers, and the passing time. Among them, attributes include:

- Gender (male or female)
- Fare checking ways (Card or Code)
- With or without-bag

The collected data are shown in Table 2.

**Table 2:** Collected passenger data.

With or without-bag	Gender	Number
With bag	male	50
	female	41
Without-bag	male	237
	female	200
Fare checking ways	Gender	Number
Card	male	232
	female	168
Code	male	198
	female	215

### 2.2 Analysis of passenger behavior at security check area

#### 2.2.1 Analysis of passenger micro-behaviors

The subway station security facilities include X-ray machines, metal detectors, luggage checking machines, and other equipment used to detect prohibited or dangerous items carried by passengers. In addition, security personnel perform handheld item checks and personal checking. Passengers are required to scan and inspect their personal belongings and carry-on luggage before entering the security checkpoint.

The time passengers take to pass through the subway station security checkpoint often depends on factors such as whether they are carrying luggage and is also affected by parameters of the security facilities, such as the speed of the security conveyor belt and the length of the security area. During the process of passing through the security facilities, the pace of passengers is not only related to their gender, but also closely related to whether they are carrying items that require checking by the security X-ray machine.

During the morning rush hour, when the subway stations are operating, due to daily commuting and other reasons, passengers often walk at a fast pace. Consequently, security personnel may not inspect passengers’ belongings at the security checkpoints. There is also a phenomenon where passengers open their messenger bags or handbags for security personnel to glance at as they pass through. This results in a situation where the time needed to pass through the security facilities is similar for passengers without luggage and those carrying messenger bags, shoulder bags, waist packs, and handbags. However, passengers with backpacks must place their luggage into the X-ray machine for checking. Even after quickly passing through the metal detector gate, they still need to wait at the rear of the X-ray machine. Therefore, there is a significant difference in the service time required for passengers carrying backpacks and suitcases compared to those without luggage or carrying a messenger bag when passing through the security facilities.

#### 2.2.2 Passenger security check time distribution

When passing through the subway station’s security facilities, passengers often need to undergo luggage checks using X-ray machines, manual inspections, and other procedures. Therefore, passengers will voluntarily queue up in an orderly manner when passing through the security facilities. During the morning rush hour, the passenger flow is at oversaturation. Under these conditions, passengers’ passage speed is faster, and those with luggage will choose manual checking to pass through the security facilities.

**Fig 2:**
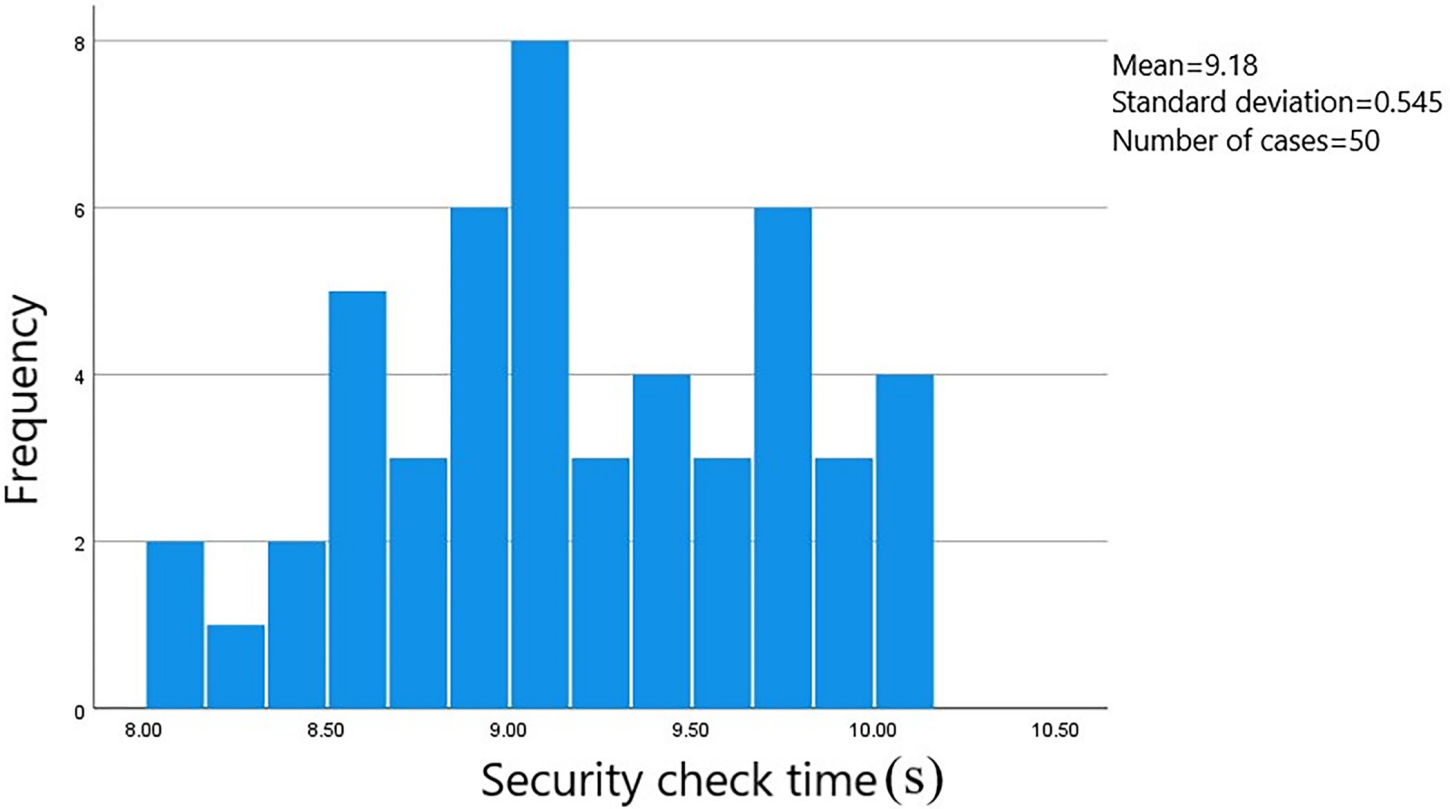
Security check time for male passengers carrying luggage.

**Fig 3:**
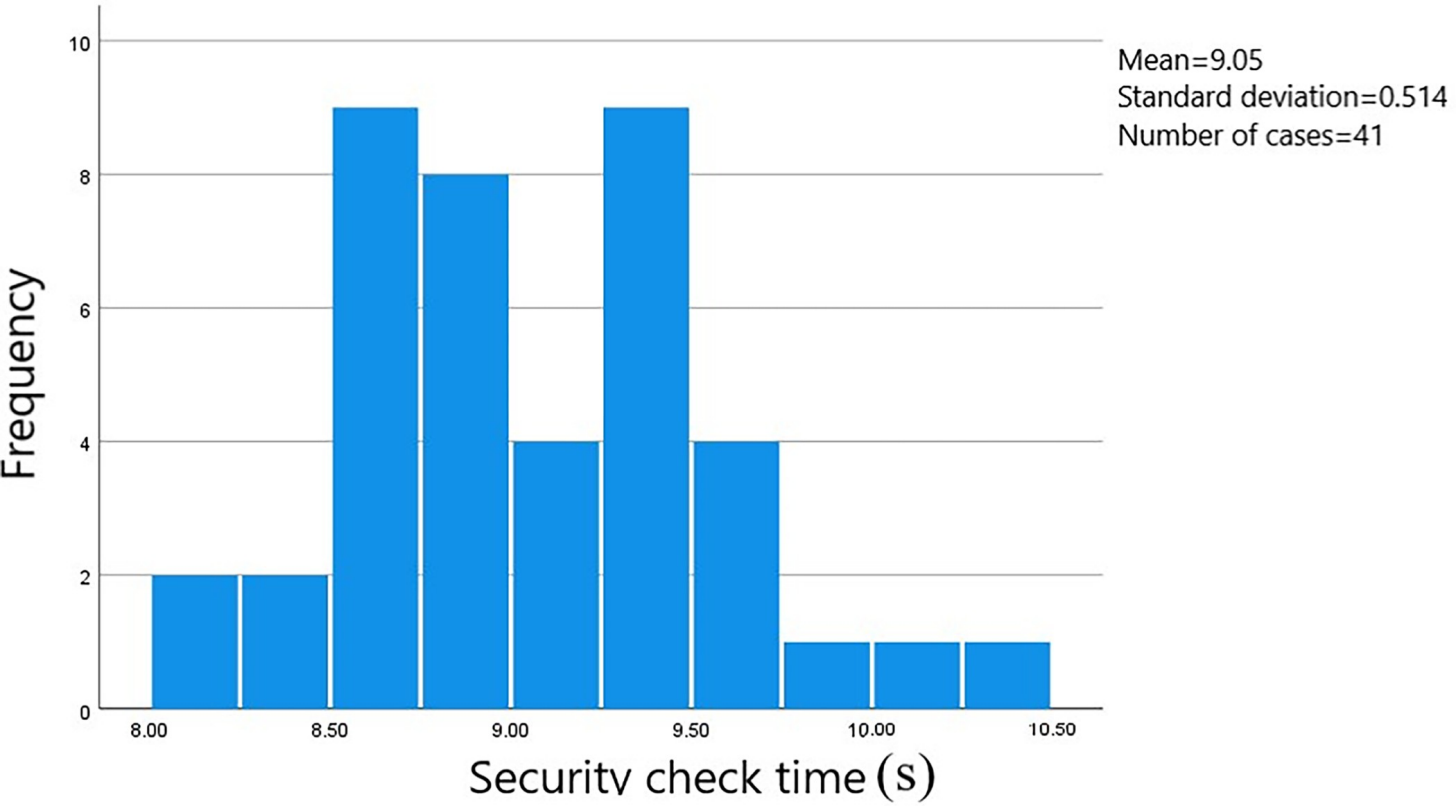
Security check time for female passengers carrying luggage.

**Fig 4:**
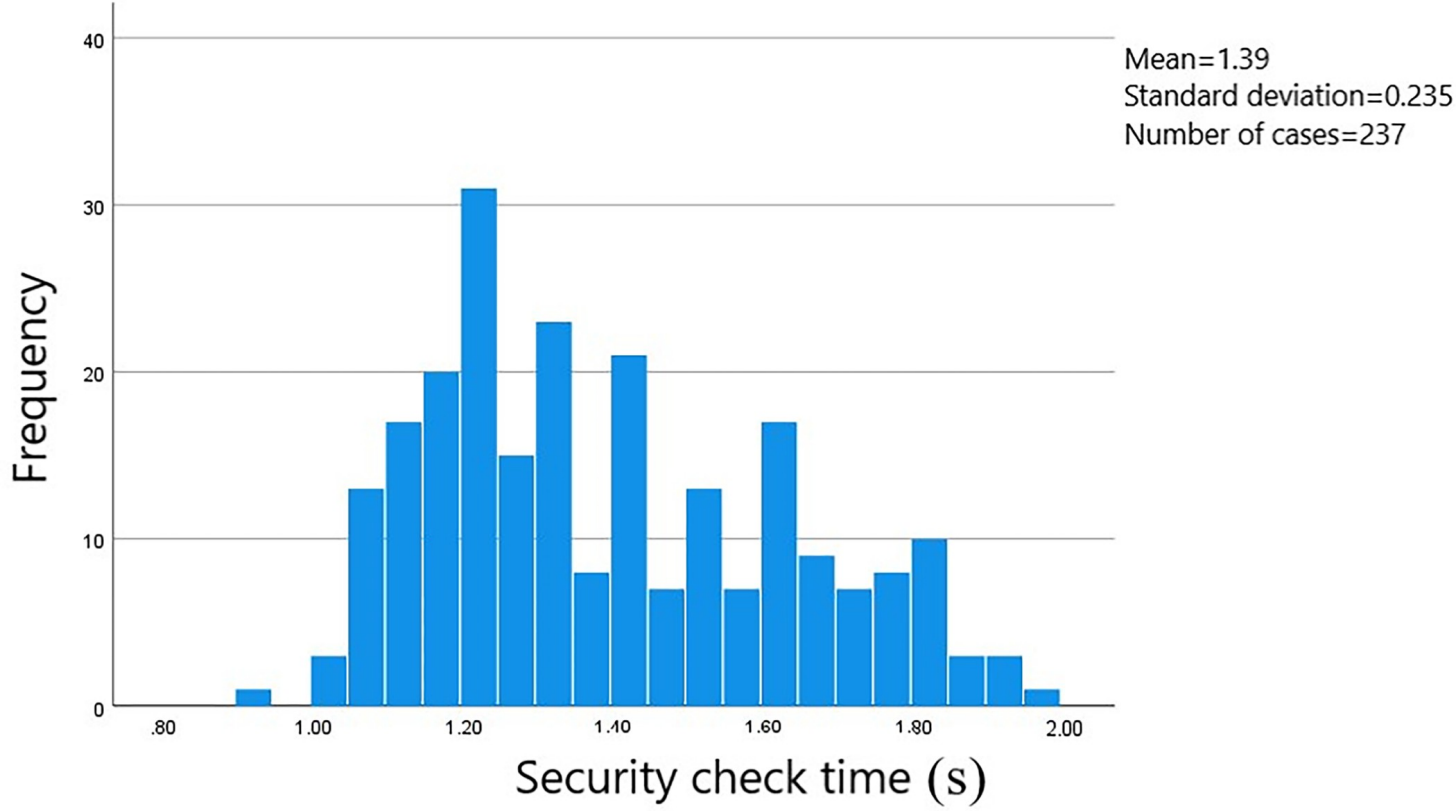
Security check time for male passengers without luggage.

**Fig 5:**
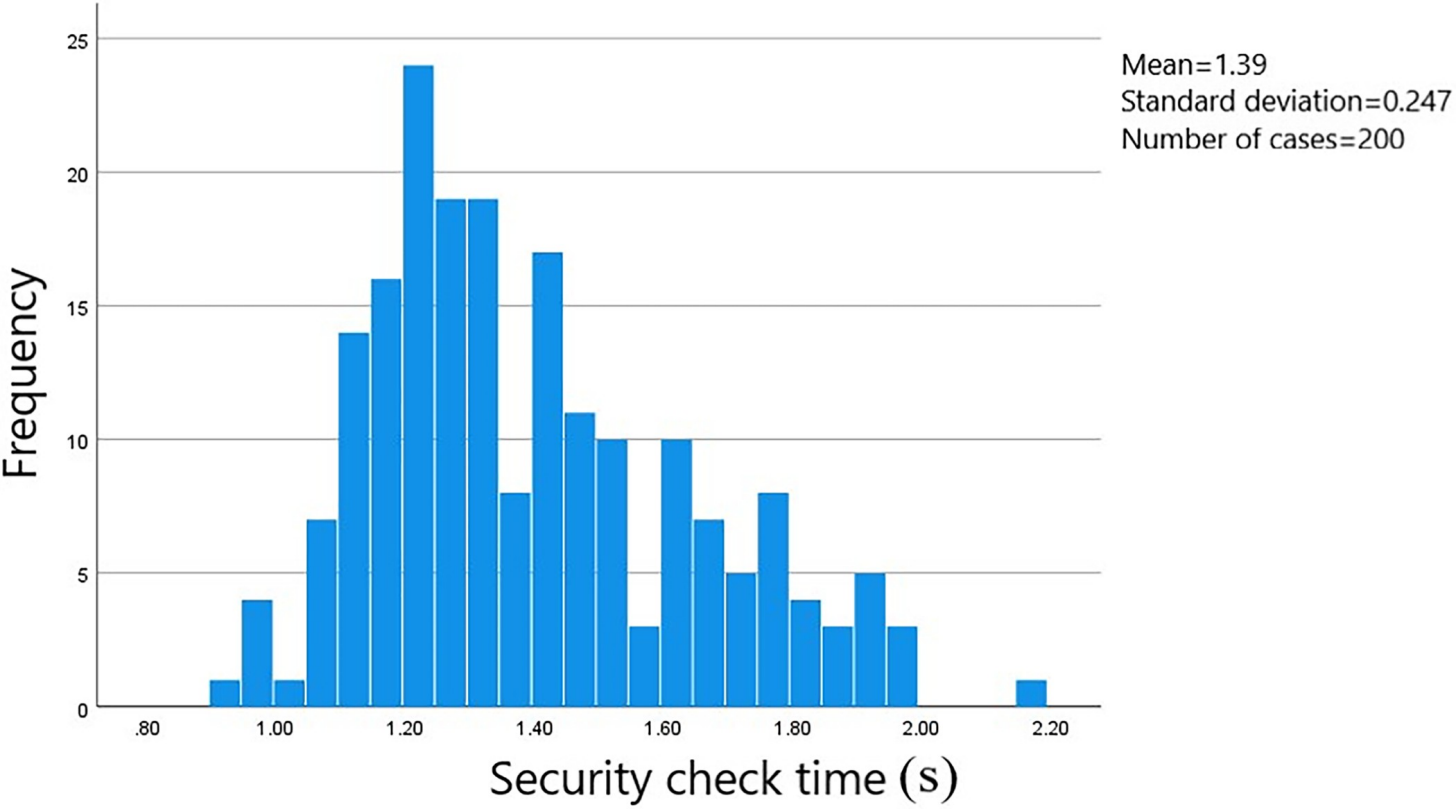
Security check time for female passengers without luggage.

A total of 528 sets of data on passenger transit times through the security check area were gathered. The starting point was when the passenger initiated manual checking or entered the security gate. The endpoint was when the passenger walked through the designated area on the security check zone floor. The data was processed using SPSS software to generate a frequency histogram of the distribution of security check times. The measured distance at the survey site for the security check area was 1.5 meters. Passengers were categorized based on whether they were carrying luggage and their gender. The time distribution is shown in Figs 2–5.

The average security check time for male passengers carrying luggage is 9.08 seconds, with the check times concentrated in the range of 8.50 to 10.00 seconds and a standard error of 0.077. The average security check time for female passengers carrying luggage is 9.15 seconds, with the check times concentrated in the range of 8.50 to 9.75 seconds and a standard error of 0.015. The average security check time for male passengers not carrying luggage is 1.39 seconds, with the check times concentrated in the range of 1.05 to 1.80 seconds and a standard error of 0.080. And the average security check time for female passengers not carrying luggage is 1.39 seconds, with the check times concentrated in the range of 1.10 to 2.00 seconds and a standard error of 0.017.

#### 2.2.3 Analysis of factors affecting passenger security check time

The study utilized on-site survey in the form of video data collection to obtain passenger demographic information related to their passage through security checkpoints. The independent variables included passenger gender and whether they were carrying luggage, while the dependent variable was the passage time through the security checkpoint. Conducted correlation analysis to examine the relationship between passenger attributes and the security check time. The data was processed using SPSS software for Pearson correlation test, and the results of the correlation analysis are presented in Table 3.

**Table 3:** Correlation analysis of passenger security check time.

Relevance	Whether carrying luggage	Gender
Pearson correlation	0.998**	-0.008
Significance (two-tailed)	0.000	0.862
Number of cases	528	528

From the table, it can be observed that the correlation between the time taken by passengers to pass through the security checkpoint and the variables of carrying luggage and gender are 0.998 and -0.008, respectively. The Pearson correlation for the variable of carrying luggage is close to 1, with a significance level of 0.01, indicating a significant correlation. On the other hand, the Pearson correlation for the gender variable is close to 0, and its significance level is not at the 0.01 level, indicating an insignificant correlation. Therefore, the study indicates that the passage time through the security checkpoint is mainly associated with the attribute of whether the passengers are carrying luggage.

The time passengers take to pass through the security checkpoint is significantly correlated with the attribute of carrying luggage, while the correlation with the gender attribute is less pronounced.

### 2.3 Analysis of passenger behavior at the security gate

#### 2.3.1 Analysis of passenger micro-behaviors

After passing through the security checkpoint at the subway station, passengers travel a distance through a corridor to reach the ticket checking gate area. Along this corridor, passengers often have to place their luggage that just passed through the X-ray machine and retrieve the items for ticket checking (such as NFC or scanning codes using their phones). Otherwise, they often end up taking out their phones and adjusting the scanning page within the gate service area, hindering the passage of subsequent passengers.

Upon entering the ticket checking area, passengers go through two stages: gate selection stage and passage stage. During the gate selection stage, due to morning rush hour characteristics, and the desire to board the subway quickly, passengers tend to choose gates that are closer to them or have shorter queue lengths for passage. During the passage stage, passengers walk while preparing to check tickets by scanning or swiping their cards. After the gate opens, they pass through quickly.

Unlike passing through the security checkpoint, where there are restrictions due to manual checking by security personnel, and as subway stations are usually crowded, passengers generally briskly pass through the ticket gates to avoid delaying other passengers. Therefore, the time it takes for passengers to pass through the ticket checking facilities is significantly influenced by the passengers’ own attributes (such as gender and whether they are carrying luggage) and the method of ticket checking methods (primarily card swiping and code scanning).

#### 2.3.2 Passenger ticketing time distribution

The ticket gate is a passenger flow control facility within the subway station, primarily responsible for checking tickets as passengers enter and exit the paid area of the subway station. The paid area of the subway station refers to the area within the station that passengers need to enter through card swiping or ticket purchasing. This area includes the station hall, platforms, and connecting passageways. Due to the narrow width of the gates and limited recognition speed, the time passengers take to prepare for ticket checking, the different methods of ticket checking used, as well as variations in gender and luggage-carrying status all affect the passenger’s ticketing time.

**Fig 6:**
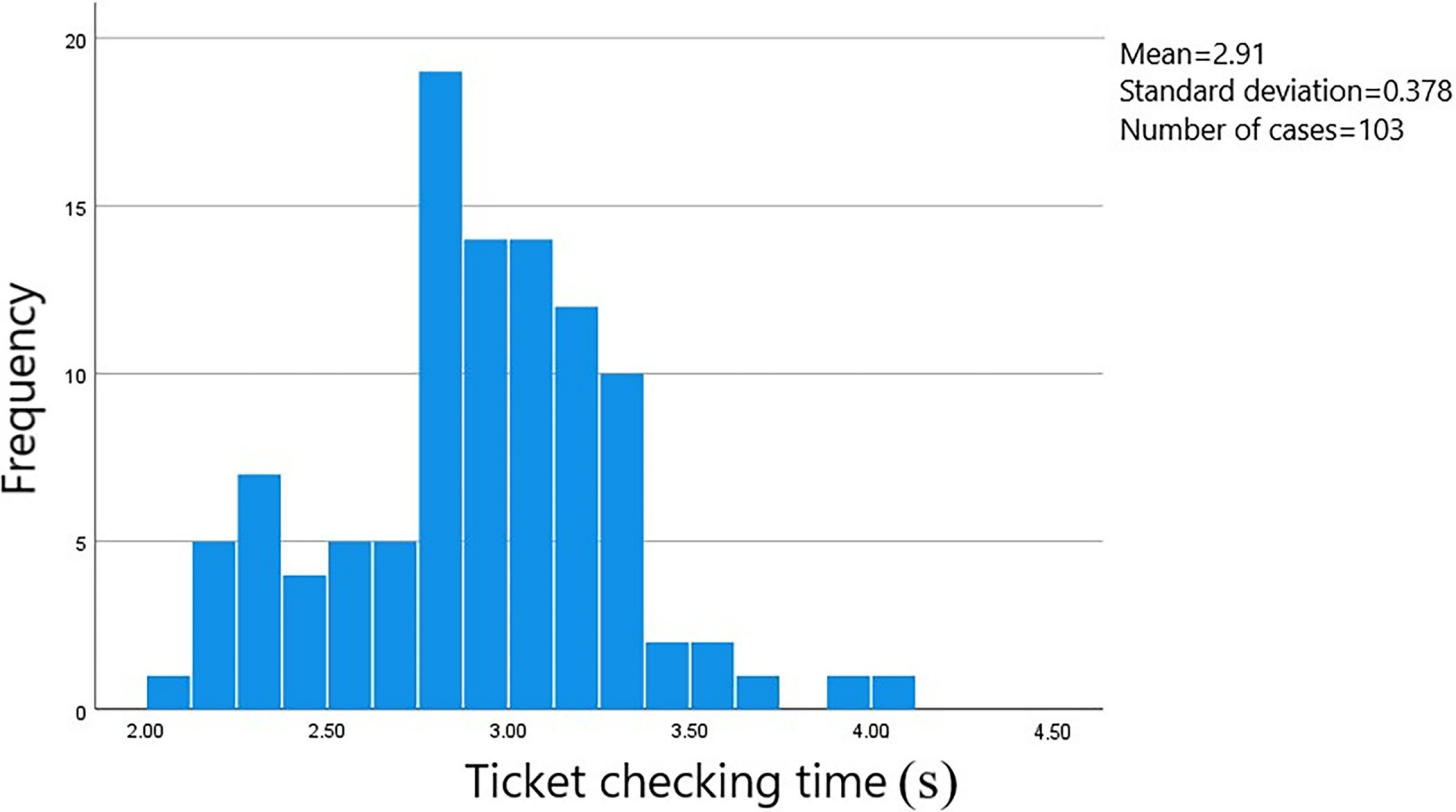
Passage time for male passengers without luggage using card swiping.

**Fig 7:**
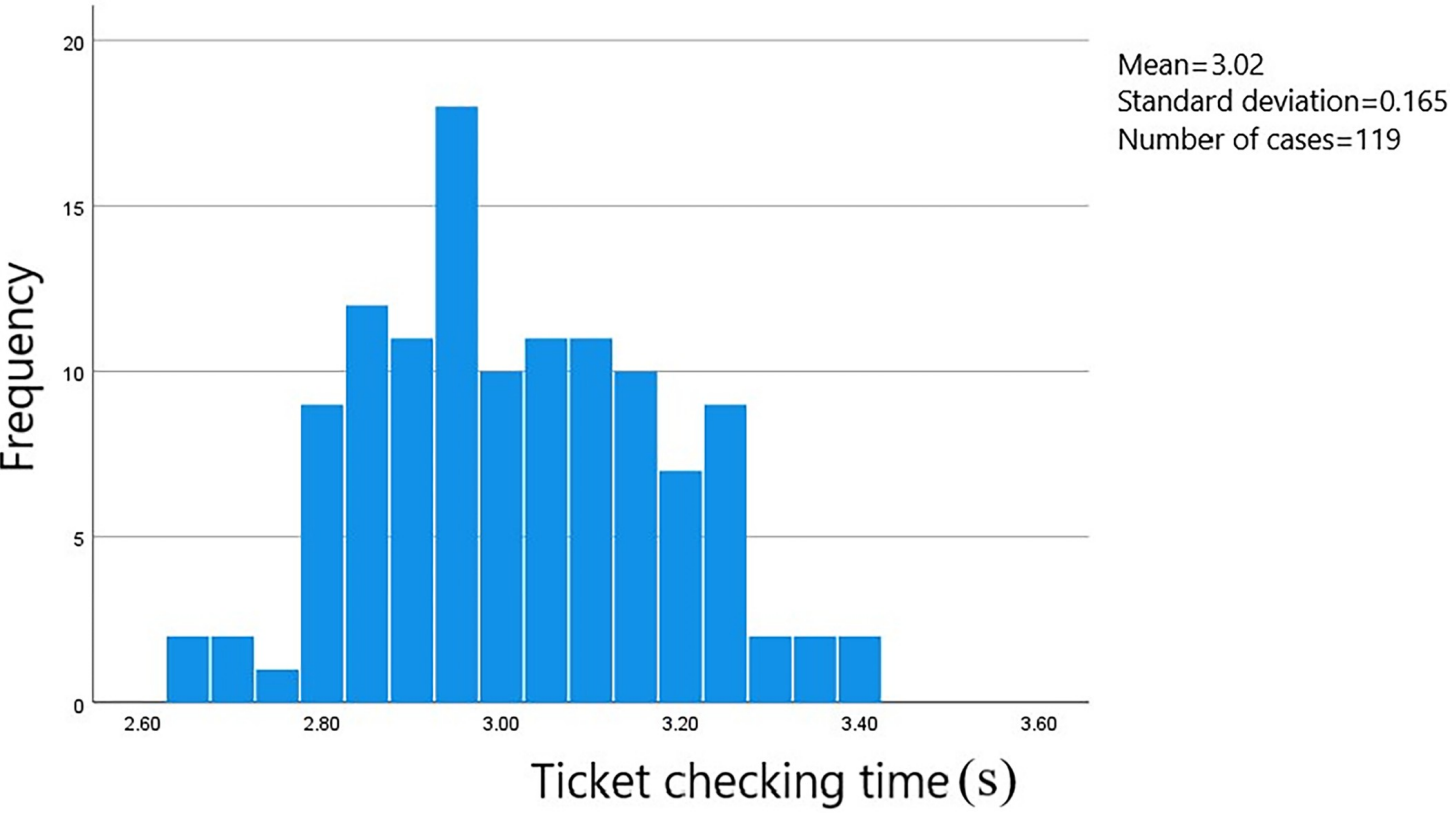
Passage time for male passengers with luggage using card swiping.

**Fig 8:**
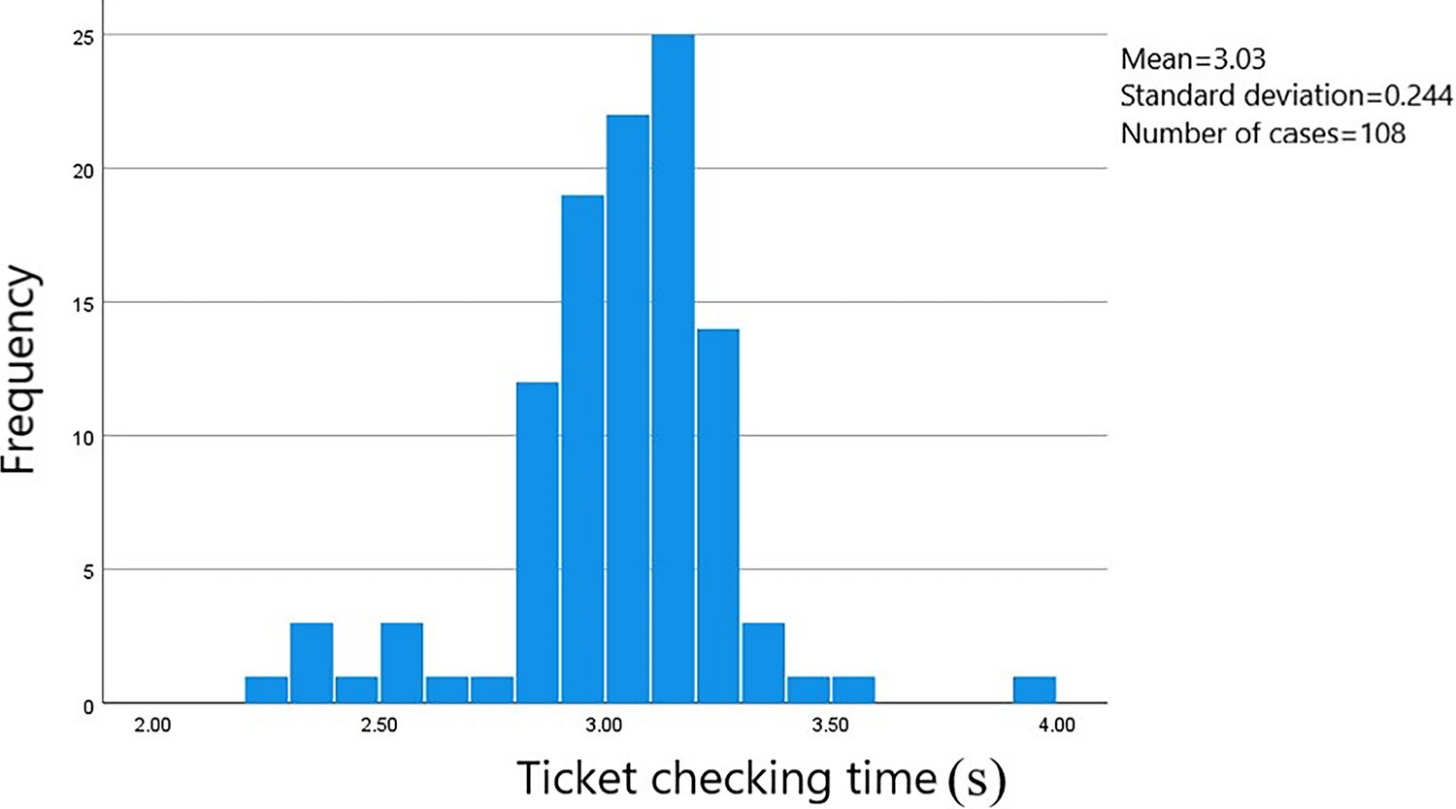
Passage time for female passengers without luggage using card swiping.

**Fig 9:**
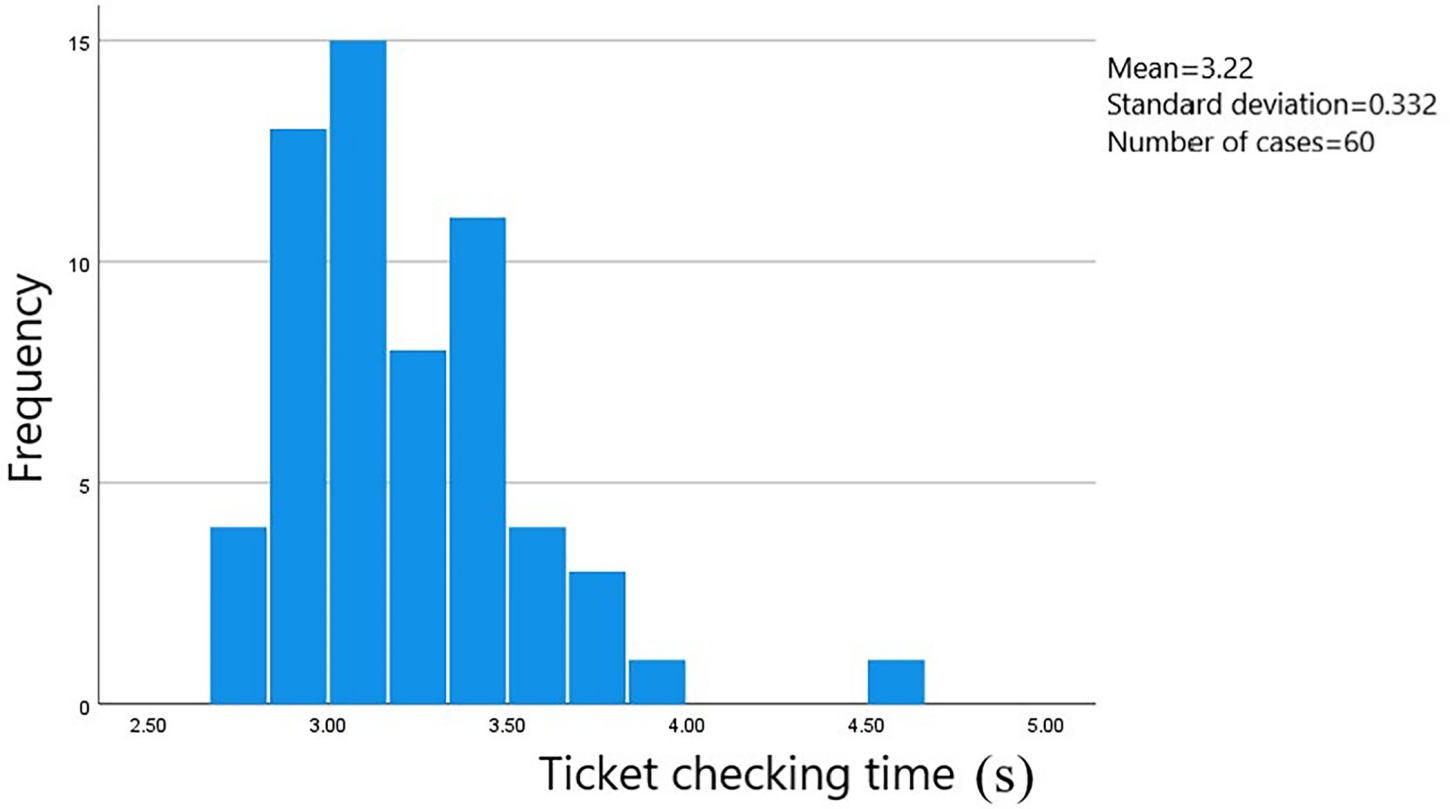
Passage time for female passengers with luggage using card swiping.

In this survey, data on the passage time of 813 groups of passengers at the ticket gates was collected. The starting point was when the passengers stopped at the ticket gate service area to begin the ticketing process, and the endpoint was when the passengers passed through the ticket gate. The data was processed using SPSS software to obtain a frequency histogram of the distribution of passenger passage times through the ticket gates. The distribution of passage times based on whether they were carrying luggage and the method of card swiping can be seen in Figs 6–9.

The average ticket-checking time for male passengers who choose to pay by card without carrying luggage is 2.91 seconds, and the security check time is concentrated within the range of 2.10 to 3.40 seconds, with a standard error of 0.037. The average ticket-checking time for male passengers who choose to pay by card and carry luggage is 3.02 seconds, and the security check time is concentrated within the range of 2.80 to 3.25 seconds, with a standard error of 0.015. The average ticket-checking time for female passengers who choose to pay by card without carrying luggage is 3.03 seconds, and the security check time is concentrated within the range of 2.80 to 3.30 seconds, with a standard error of 0.023. And the average ticket-checking time for female passengers who choose to pay by card and carry luggage is 3.22 seconds, and the security check time is concentrated within the range of 2.80 to 3.50 seconds, with a standard error of 0.042.

#### 2.3.3 Analysis of the causes of passenger passage time at the gate machine

The study employs on-site investigation at subway stations to obtain passenger demographic data through video surveys of ticket gate facilities. Correlation analysis is conducted on the dependent variable of the time spent passing through ticket gate facilities and the independent variables, passenger gender, luggage-carrying status, and ticket-checking methods, in order to establish the relationship between passenger attributes and ticket-checking time. The Pearson correlation test is used to process the data in SPSS software, and the results of the correlation analysis are shown in Table 4.

**Table 4:** Correlation analysis of passenger ticket-checking time.

Relevance	Whether carrying luggage	Gender	Ticket checking method
Pearson correlation	0.464	0.162	0.461
Significance (two-tailed)	0.000	0.000	0.000
Number of cases	813	813	813

The table shows that Pearson correlation coefficients between the time taken for passengers to pass through the ticket gate facilities and the attributes of carrying luggage, gender, and ticket-checking methods are 0.464, 0.162, and 0.461, respectively, indicating a positive correlation. With a significance level at 0.01, the correlations are all significant. Thus, the study indicates that the passage time of passengers at the ticket gate is mainly correlated with three attributes: whether the passenger is carrying luggage, gender, and the method of ticket checking.

## 3 Passenger behavior labeling

### 3.1 Software selection for simulation

AnyLogic is a simulation software based on social force modeling, commonly used in traffic flow optimization and urban planning. This software supports discrete event, system dynamics, and cellular automaton modeling methods, allowing users to establish dynamic and complex models for system research. Through its simulation functionality, it enables testing of different decision scenarios and trend prediction.

As a modeling and simulation platform, AnyLogic includes libraries such as the pedestrian library and the rail library. The pedestrian library enables the simulation of personnel movement and interaction, consisting of environment modeling and behavior modeling. It integrates path planning algorithms and obstacle detection functions to prevent collisions. Utilizing the AnyLogic pedestrian library ensures the validity of simulating pedestrian travel within the concourse level of a rail transit station.

According to this software, we can build the physical model of subway station, which includes environmental factors such as behavioral boundaries, obstacles, and starting lines, and basic service facilities such as SC facilities, TC facilities, and channel-type facilities. After that, the logic model is established, which refers to the construction of logic relationship to control pedestrian behavior (e.g., pedestrian generation, pedestrian movement, pedestrian waiting, pedestrian selection output, pedestrian annihilation, etc.) and train behavior (e.g., train arrival, train movement, train departure, etc.). Therefore, we need to calibrate pedestrian behavior.

We run the simulation in Intel(R) core(TM) i5-10210U 1.60GHZ PC with 4 GB memory, and we input all the simulation parameters of the model into Anylogic Professional 8.7.0 software with JAVA2.0.

### 3.2 Passenger behavior calibration

As we mentioned before, we studied the time required for passengers with different attributes to pass through security and ticket checking facilities in the subway station, categorizing them into eight groups based on gender, luggage, and ticket checking method. The security and ticket checking times for each passenger group category were determined based on research results and are presented in Table 5.

**Table 5:** Average security check time and average ticket check time for passengers with different attributes.

category	gender	whether carrying luggage	ticket checking method	average security check time/s	average ticket checking time/s
1	Male	Without luggage	NFC	1.39	2.91
2	Male	With luggage	NFC	9.08	3.02
3	Female	Without luggage	NFC	1.39	3.03
4	Female	With luggage	NFC	9.15	3.22
5	Male	Without luggage	QR code	1.39	3.26
6	Male	With luggage	QR code	9.08	3.37
7	Female	Without luggage	QR code	1.39	3.13
8	Female	With luggage	Scan QR code	9.15	3.49

**Fig 10:**
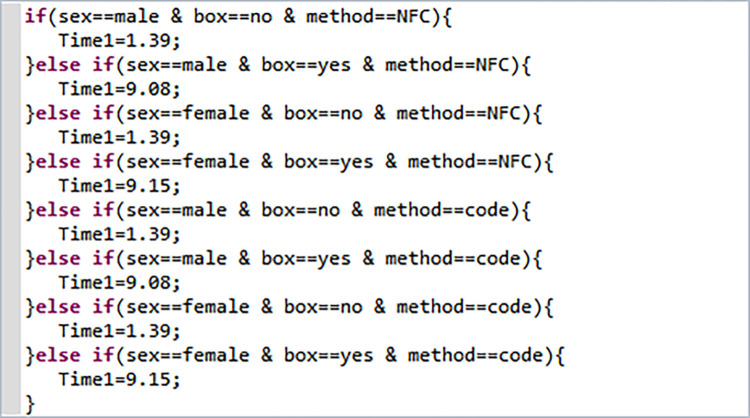
Function for security check time.

**Fig 11:**
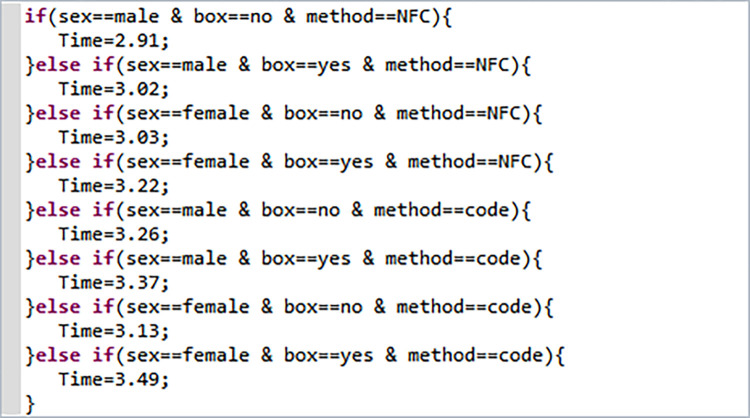
Function for ticket check time.

The functional form for calibrating the security check and ticket check times for different categories of passenger groups is shown in the Figs 10 and 11.

## 4 Data analysis

### 4.1 Station analysis

#### 4.1.1 Ciqunan station overview

**Fig 12:**
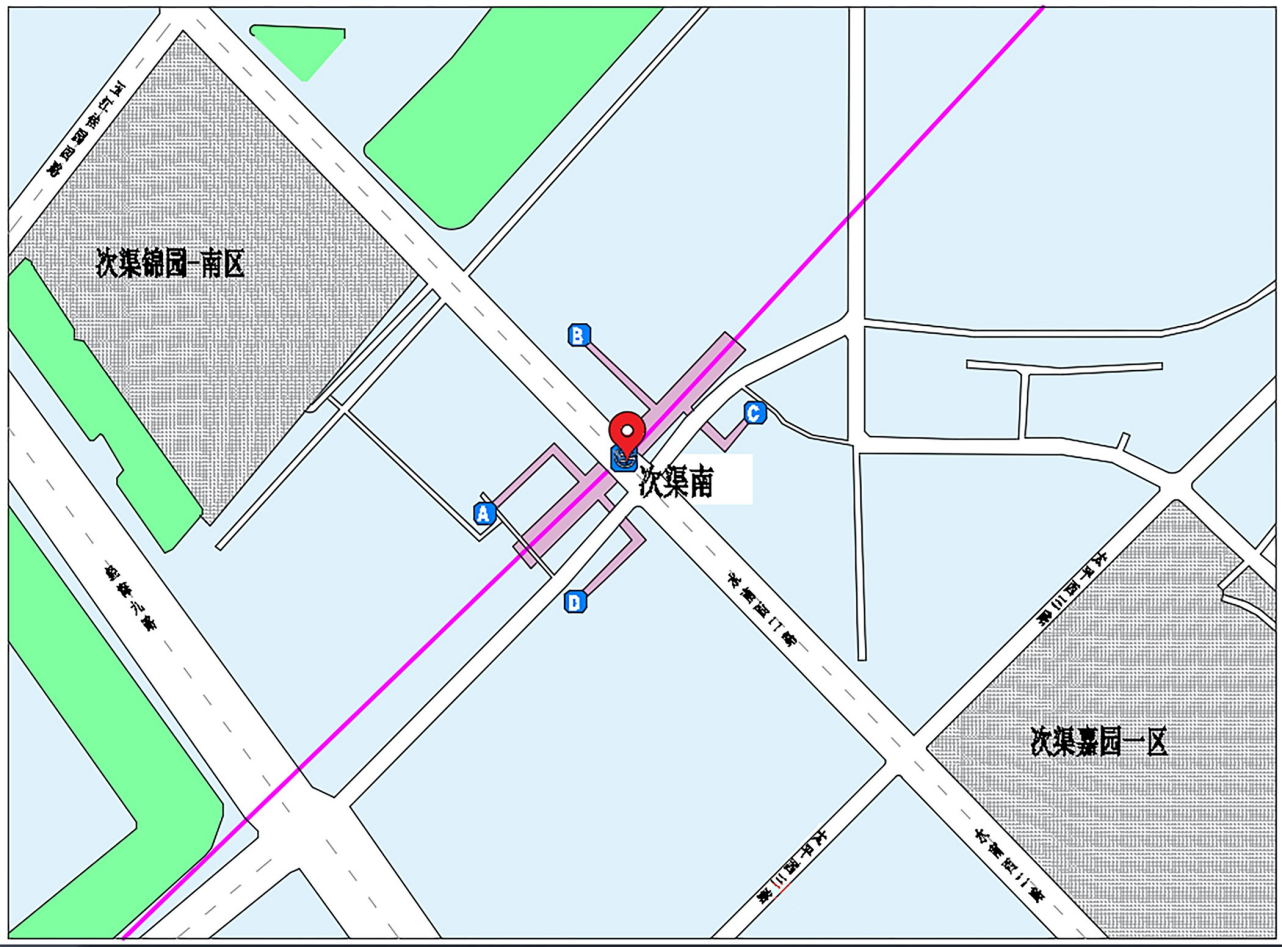
Overview of the geographical location of Ciqunan Station.

Ciqunan Station (Fig 12) is situated at the intersection of Puxi Road and Tongxiang Street in Tongzhou District, Beijing, China. It is managed by Beijing MTR Corporation Limited and serves the Beijing Subway’s Yizhuang Line. The station has a total construction area of 11,273 square meters, with a main building area of 8,603 square meters and a total length of 208.85 meters. It features four exits: A, B, C, and D.

The peak entry passenger flow of rail transit refers to the volume of passengers waiting to enter the subway station during peak hours, exhibiting obvious spatiotemporal characteristics. The area surrounding the station comprises large residential communities with significant commuting populations, such as Ciqu Jiayuan East to the southeast and Ciqunanli to the north, among others. Tidal commuting features are particularly prominent, with the morning peak commuting passenger flow being the main component on weekdays, characterized by short duration, concentration, and speed. Passengers have clear travel purposes and often pass through security checks and ticket gates rapidly, proceeding to the platform level for waiting. During the morning peak period, entrances B and C of Ciqunan Station experience a large proportion of the total passenger flow, leading to pronounced queuing in the ticket-checking and security areas in that direction.

#### 4.1.2 Ciqunan station layout

**Fig 13:**
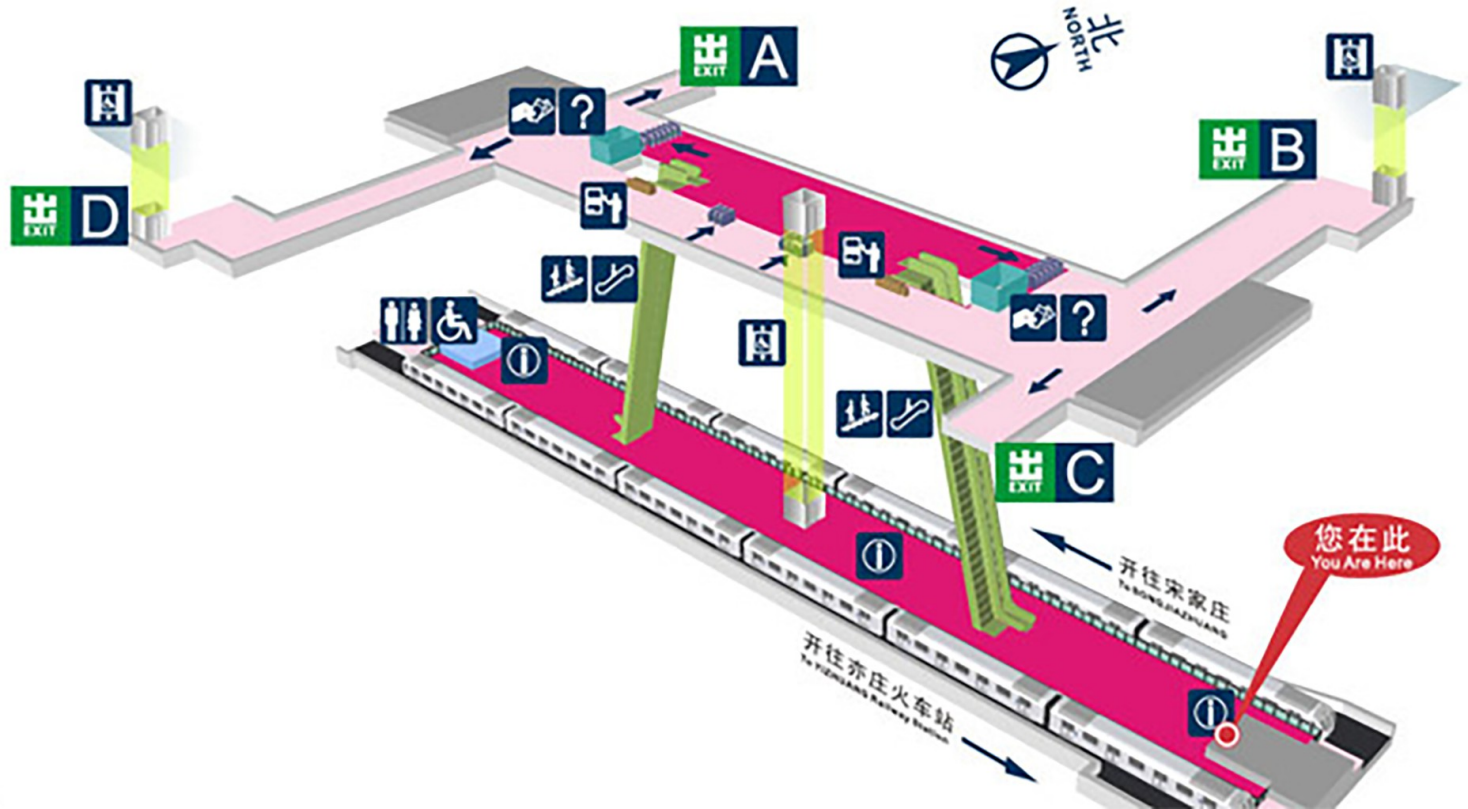
Layout plan of Ciqunan Station.

Ciqunan Station is an underground, two-tier, three-span structure station. It comprises a concourse level, divided into a paid area in the middle and unpaid areas at both ends, and a platform level with an island platform (Fig 13).

Passengers entering the station pass through security and ticketing facilities before choosing stairs or an elevator to access the platform level from the concourse level. Exiting passengers use stairs, escalators, or elevators to transition from the platform level to the concourse level and proceed through the ticket gates to exit the station. In the western direction of Ciqunan Station (entrances A and D), there is one security gate and one X-ray machine, along with two ticket gates. In the eastern direction (entrances B and C), there are two security gates, one X-ray machine, and four ticket gates, with three gates for entry and one for exit, all used for entry during the morning peak hours.

#### 4.1.3 Passenger flow forecast for Ciqunan Station

The passenger flow forecast is obtained through on-site video data investigation. Surveying the passenger flow at Ciqunan Station during the morning peak hours from Monday, April 17th to Friday, April 21st, the passenger flow at the security check and ticket checking facilities areas was recorded and tallied. The total inflow of passengers during peak hours was calculated to be 2520 passengers per hour, with entrance A receiving 300 passengers per hour, entrance B 840 passengers per hour, entrance C 1140 passengers per hour, and entrance D 240 passengers per hour.

Following the acquisition of the video footage, a statistical analysis of passenger characteristics was carried out, involving a total of 1167 individuals. Among them, there were 607 males and 560 females; 969 individuals were without backpacks, while 198 were carrying backpacks. Additionally, 560 passengers used the card-swiping (NFC) ticket checking method, while 607 chose the mobile phone QR code scanning method. Subsequently, the collected passenger flow data was classified into eight categories based on gender (male, female), luggage carriage (with or without), and ticket checking method (card-swiping, QR code scanning). The proportions of different passenger groups within the passenger flow are presented in the Table 6.

**Table 6:** Proportion of passengers with different attributes at Ciqunan Station.

Total passenger flow	male-female ratio	percentage of passengers carrying luggage (no luggage/with luggage)	ticket checking method (Swipe card NFC/ Scan QR code)
2520 PEOPLE PER HOUR	0.52/0.48	0.83/0.17	0.48/0.52

The peak hour influx at Ciqunan Station is approximately 2520 passengers per hour, with the majority of the morning peak flow entering the subway station through entrances B and C. This paper primarily analyzes the impact of the current morning peak hour passenger flow on Ciqunan Station, as well as the effect after optimizing the quantity of security check and ticket checking facilities.

Based on the aforementioned data, a simulation scenario will be established in AnyLogic.

#### 4.1.4 Simulation analysis

**Fig 14:**
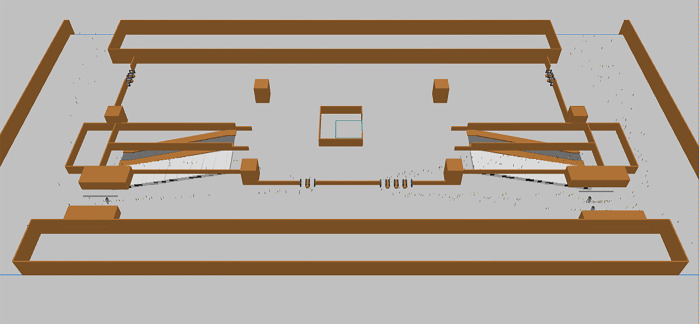
3D schematic diagram of the current status simulation on the concourse level.

**Fig 15:**
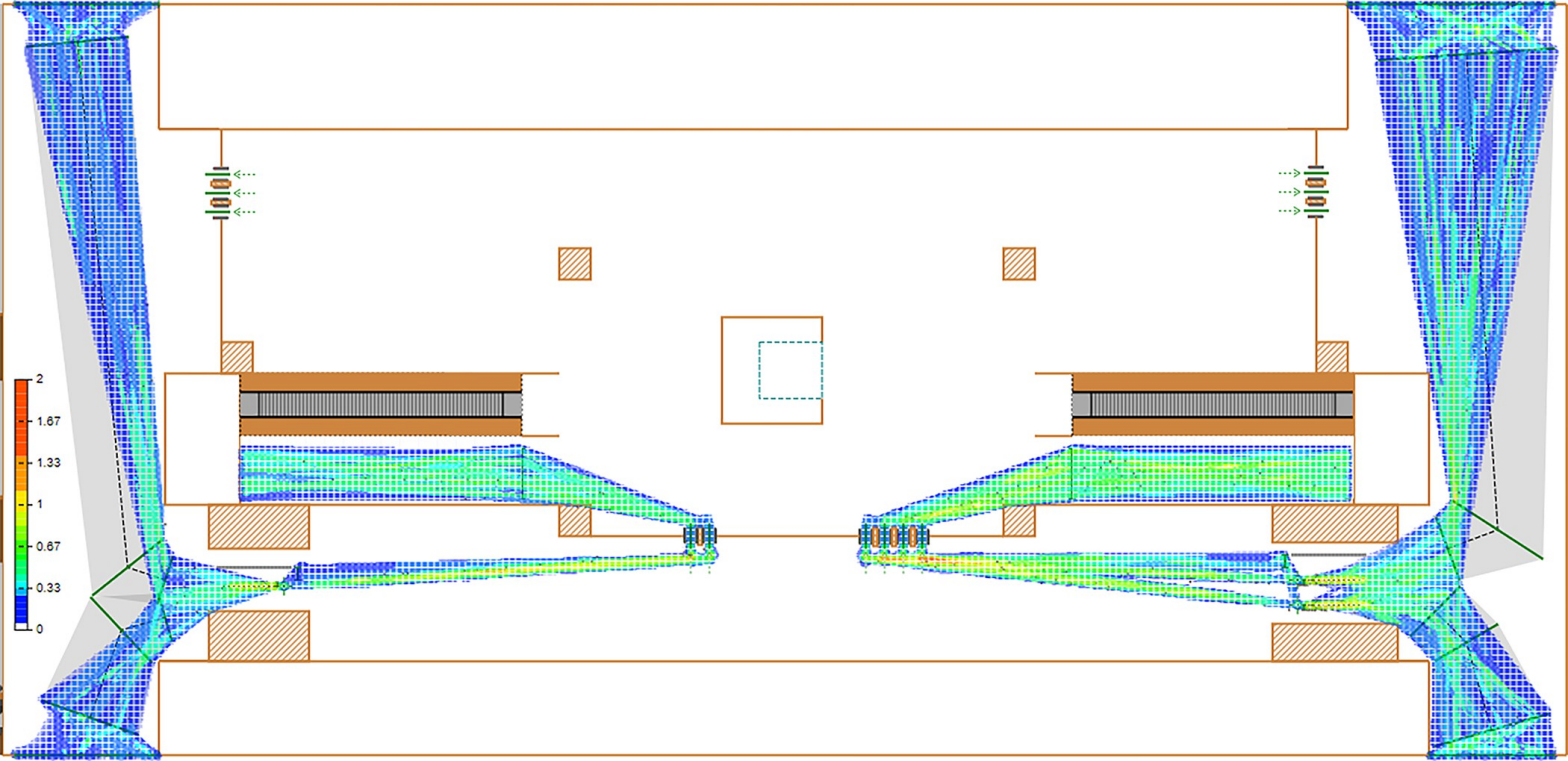
Current density map of passenger flow at Ciqunan Station.

Execute the current status simulation model for Ciqunan Station. Through the 3D animation (Fig 14), it can be observed that passengers, due to their gender, whether they are carrying luggage, and the method of ticket checking, have different attributes, and experience varying delay times at the security check and ticketing facilities. After running for more than 10 minutes, a mixed pedestrian flow of passengers with and without luggage passes through the security check facility, leading to a gathering of people in the bottleneck area in front of the security check facility and the appearance of queues, which take some time to dissipate. Passengers in the ticketing facility area pass through normally, but the density of the passenger area in front of the ticket checking facility on the east side is relatively high (Fig 15).

In the early peak hours (7:30–8:00), individual entry and exit service facility times were recorded to compute the system dissipation time. During this period, the system dissipation time for passengers entering the station and passing through the security check and ticketing facilities on the west side was 31.56 minutes, while the system dissipation time on the east side was 36.12 minutes.

**Fig 16:**
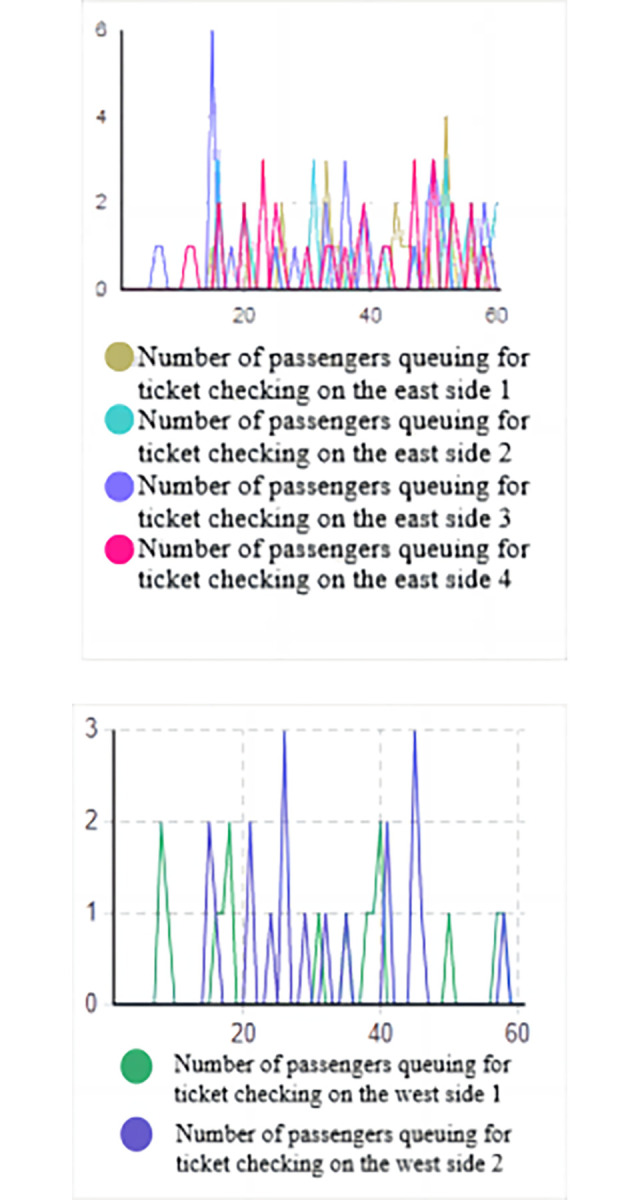
Simulation result graph of the number of people queuing for ticket checking. (a) Number of passengers queuing for ticket checking on the west side of the entrance. (b) Number of passengers queuing for ticket checking on the east side of the entrance.

**Fig 17:**
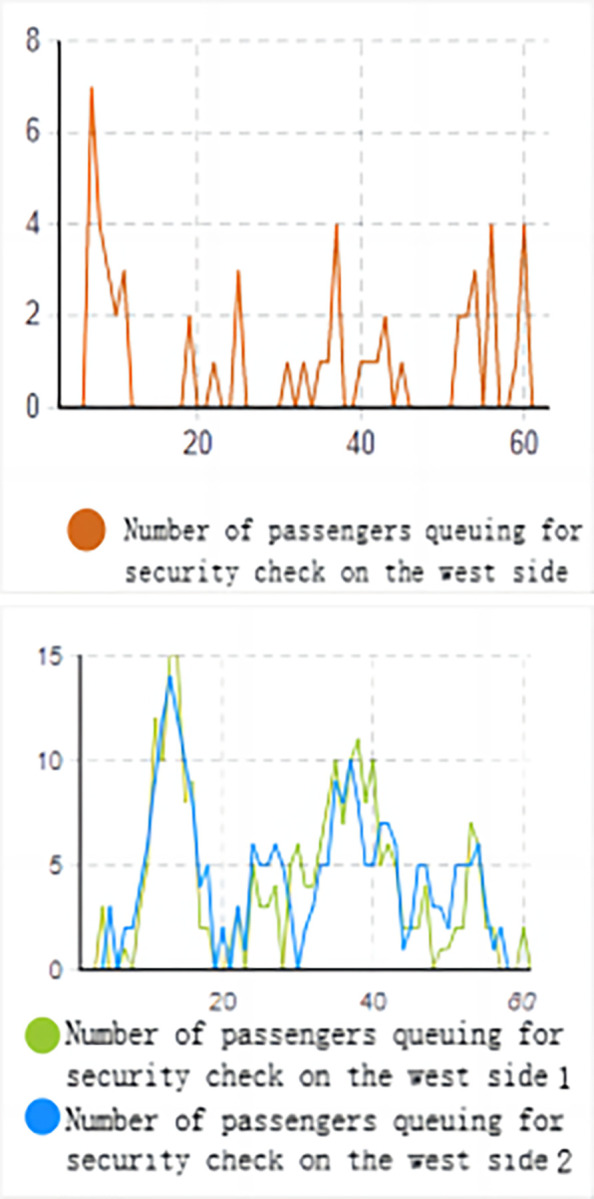
Simulation result graph of the number of people queuing for security check. (a) Number of passengers queuing for security check on the west side of the entrance. (b) Number of passengers queuing for security check on the east side of the entrance.

Based on the data from the above figure (Figs 16 and 17), it is evident that without implementing optimization measures, the high volume of passenger traffic during the early peak hours results in a high passenger density in the security check channels on both the west and east sides, as indicated by the heat density map.

On the east side, the typical queue length for ticketing facilities is 1 to 4 people, with a maximum queue length of 8 people. On the west side, the typical queue length for ticketing facilities is 1 to 2 people, with a maximum queue length of 3 people. The queue length for security check facilities is higher. On the west side, the typical queue length for security check is 1 to 4 people, with a maximum queue length of 7 people. On the east side, the typical queue length for security check is 3 to 10 people, with a maximum queue length of 15 people. Prolonged waiting times often cause discomfort for passengers entering the station.

Based on the system dissipation time, it can be inferred that the system dissipation time for passengers on the west side is 31.56 minutes, while the passage efficiency for passengers on the east side is lower, with a system dissipation time of 36.12 minutes.

### 4.2 The current situation analysis and optimization measures

#### 4.2.1 The current situation analysis

From the heatmap of pedestrian flow, it can be observed that the passenger density forms a funnel shape in front of the security check and ticketing facilities, indicating a bottleneck area with high passenger density. The high-density area in front of the security check and ticketing facilities appears as a straight line, indicating a clear queuing phenomenon.

From the queue length-time line chart, it is apparent that the passenger flow on the east side is higher, leading to excessively long queues at the security check, with up to 15 people waiting in line, suggesting insufficient efficiency in handling passengers during peak hours. On the west side, the maximum number of people in the security check queue is 7, and the queue length is relatively long. The overall queue length in the ticketing area is shorter, indicating higher passenger passage efficiency. Additionally, there is a mixed flow of passengers with and without luggage, and the queuing and waiting of passengers with luggage at the security check area result in lower passenger passage efficiency.

From the system dissipation time, it is evident that the passenger flow on the west side can all enter the station within 30 minutes during the morning peak hours. However, the system dissipation time for the passenger flow on the east side, passing through the security check and ticketing facilities, is 36.12 minutes. This indicates that during the morning peak hours, it is not possible to promptly allow all passengers to enter the station, and congestion will affect the normal entry of passengers for some time thereafter.

In conclusion, it is recommended to optimize the layout of the security check and ticketing facilities to enhance passenger entry efficiency into the station. Furthermore, organizing the passenger flow routes to segregate passengers with luggage from thosflow routes to separate passengers with luggage from those without, in order to enhance the entry efficiency of passengers.

#### 4.2.2 Optimization measures

According to the on-site investigation, the passage width of the security check area at Ciqunan Station is 4.60m. The security check gates have a known width of 0.80m, while the security check X-ray machine measures 1.00m in width. Up to 4 security check gates can be installed. The ticketing facility area has a width of 5.50m, with ticketing gates measuring 0.80m and accessible gates measuring 1.15m. It is recommended to install at least one accessible gate, along with a maximum of 5 ticketing gates and one accessible gate. Moreover, the passenger throughput capacity of the ticketing facilities should exceed that of the security check facilities on the same side.

If movable barriers are placed at the security check area to separate passengers with luggage from those without, it is recommended to have a minimum of two security check gates on each side. Considering the magnitude of passenger flow on both the east and west sides, as well as the throughput capacity of the security check and ticketing facilities, optimization measures should be implemented for the layout and quantity of security check and t ticketing facilities at Ciqunan Station.

1. To alleviate the increased passenger throughput at the security check facilities on the east side, an additional security check gate should be installed on both the west and east sides of the subway station. To accommodate the increased passenger flow at the ticketing facilities, an additional ticketing gate should be added on the east side of the subway station.
2. In the two security check queues on the west side, movable barriers measuring 1.2m in length should be installed. On the east side, near the security check X-ray machine queue, a movable barrier measuring 2.4m in length should be placed. These movable barriers will be used to separate passengers with luggage from those without, allowing passengers with luggage to pass through the security check in an orderly manner and saving waiting time for passengers without luggage.

### 4.3 Simulation optimization

**Fig 18:**
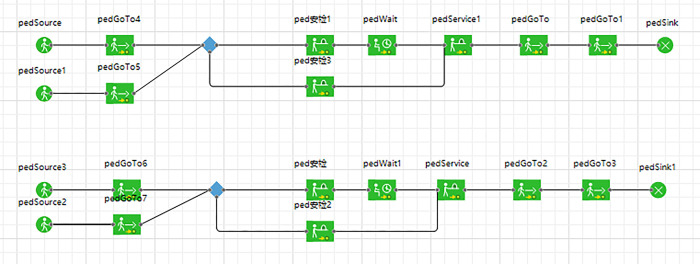
Pedestrian logic diagram after optimization.

Add movable barriers in the security check areas on both sides to separate passengers with luggage from those without. The pedestrian logic diagram needs to be adjusted, and the optimized pedestrian logic diagram is as Fig 18.

By running the optimized simulation at Ciqunan Station, it has been observed through a three-dimensional animation that the addition of movable barriers has alleviated congestion in bottleneck areas of the security check facilities. Passengers are now able to select their queues in advance and wait for service. Furthermore, segregating passengers based on whether they are carrying luggage or not has resulted in an overall reduction in waiting time for passengers without luggage. During the morning peak hours, there is a higher proportion of passengers who do not require baggage checks due to the characteristics of commuter flow. Implementing these optimization measures can enhance the overall efficiency of pedestrian flow through security checks.

**Fig 19:**
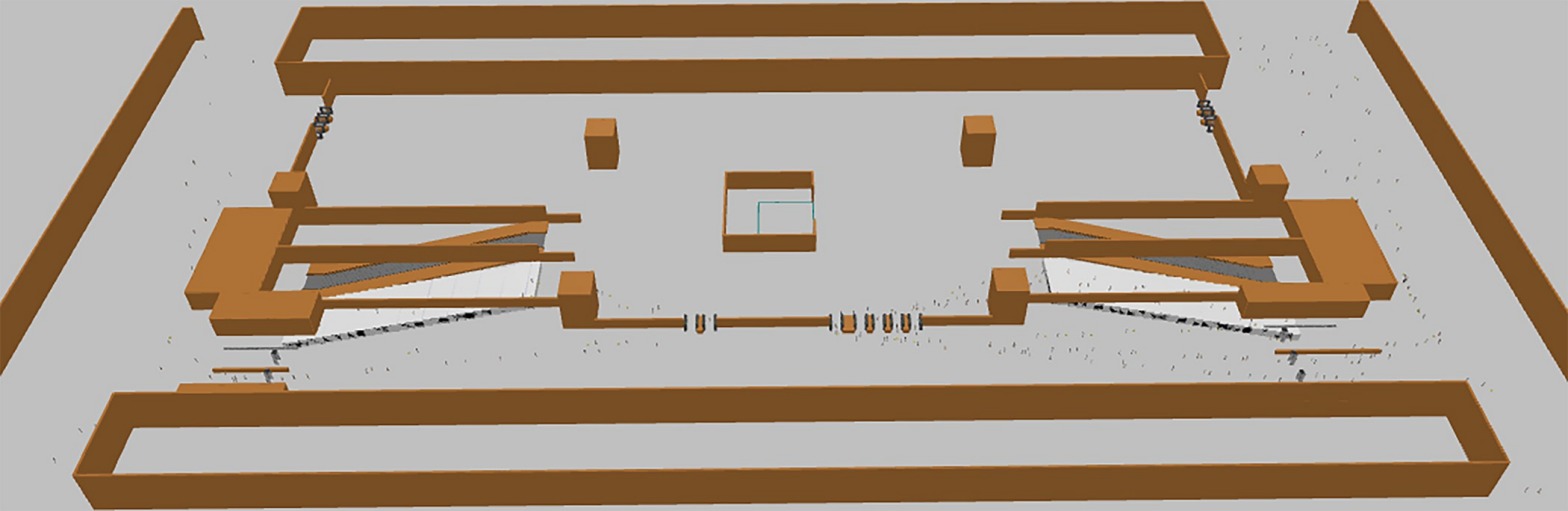
3D conceptual diagram of the simulated concourse level after optimization.

**Fig 20:**
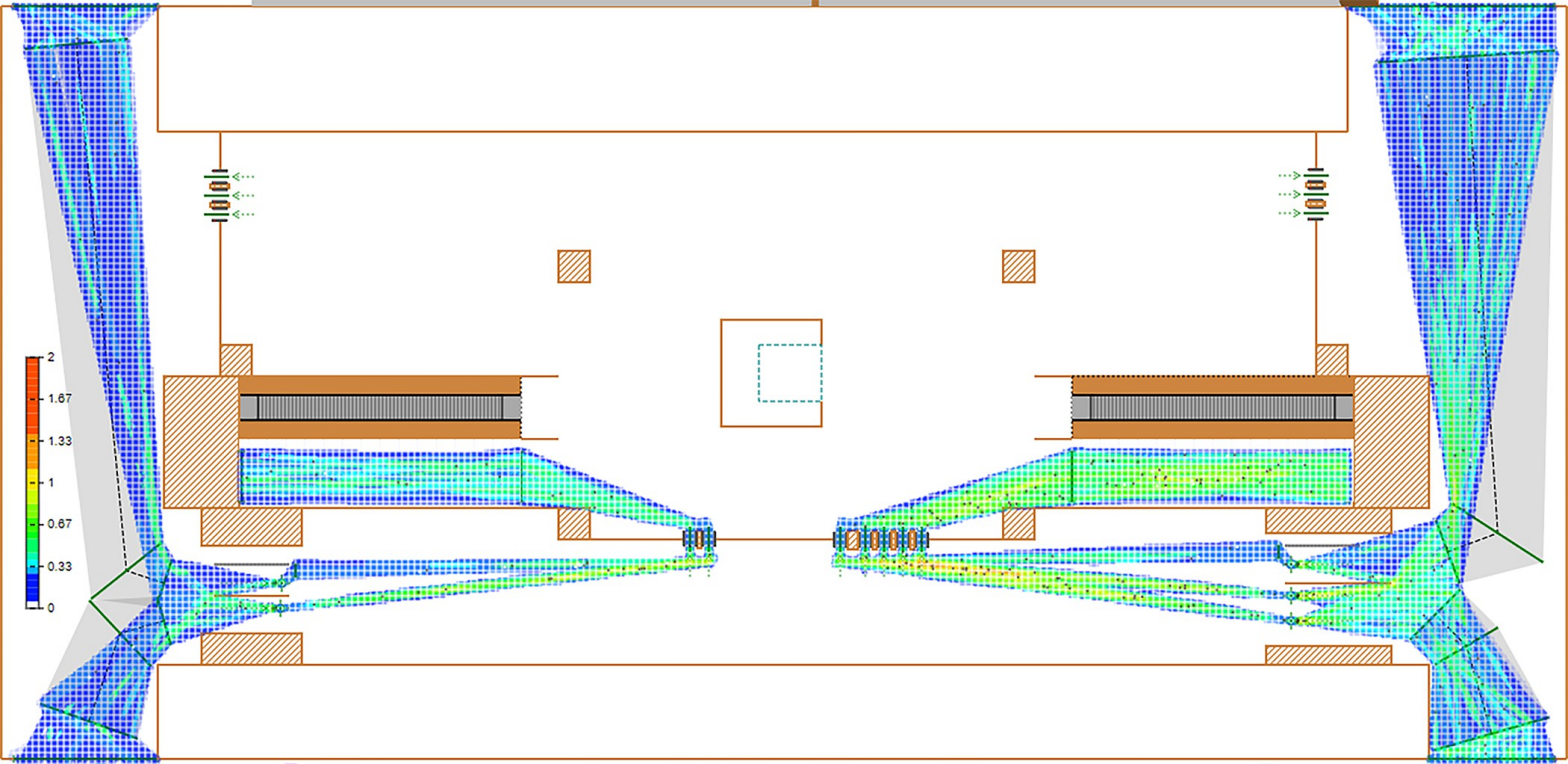
Density map of inbound passenger flow at Ciqunan Station after optimization.

An additional security gate has been installed on both the west and east sides to complement the movable barriers, segregating passenger groups and enhancing the efficiency of the security check facilities. With the improved security passage efficiency, there will be an increased flow of passengers entering the ticket checking facilities. Given the high service pressure on the ticket checking turnstiles on the east side, an additional turnstile is added to enhance the efficiency of ticket checking (Figs 19 and 20).

During the morning peak hours of 7:30 to 8:00, the dispersal time for passengers at the security check and ticket checking facilities is 30.54 minutes on the west side and 30.87 minutes on the east side.

Based on the optimized quantity of security check and ticket checking facilities, as well as the addition of activity barriers to separate passengers carrying luggage from those without, simulation experiments were conducted to obtain results. It was found that due to the early diversion of passenger flow by the barriers in the security check area, the passenger density decreased. Additionally, the addition of one ticket checking facility on the east side alleviated the passenger density pressure in the ticket checking area.

**Fig 21:**
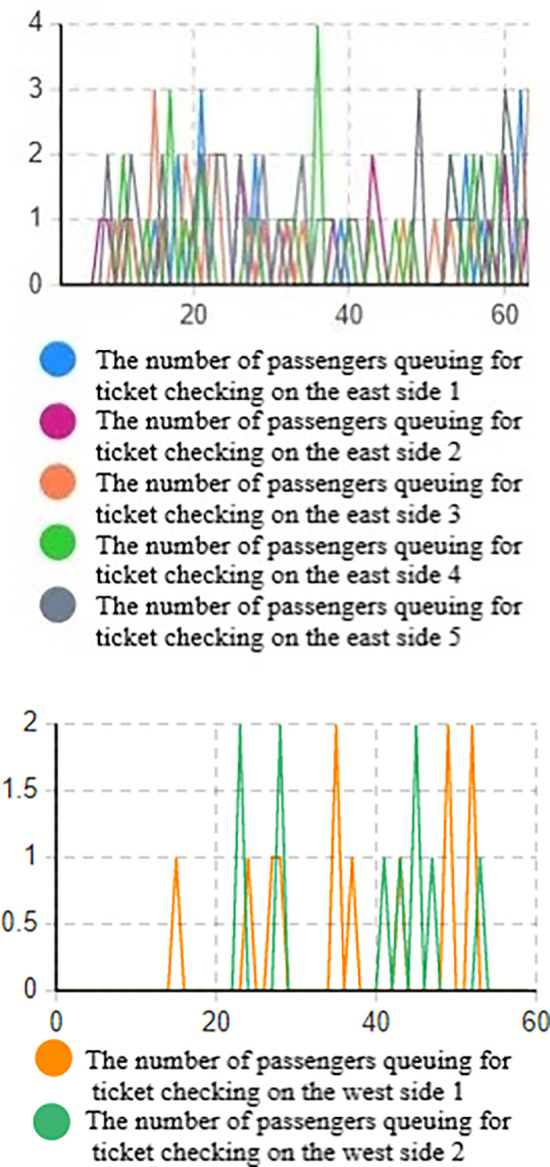
Simulation results of queueing numbers after optimization for ticket checking. (a) The number of passengers queuing for ticket checking on the west side after optimization. (b) The number of passengers queuing for ticket checking on the east side after optimization.

**Fig 22:**
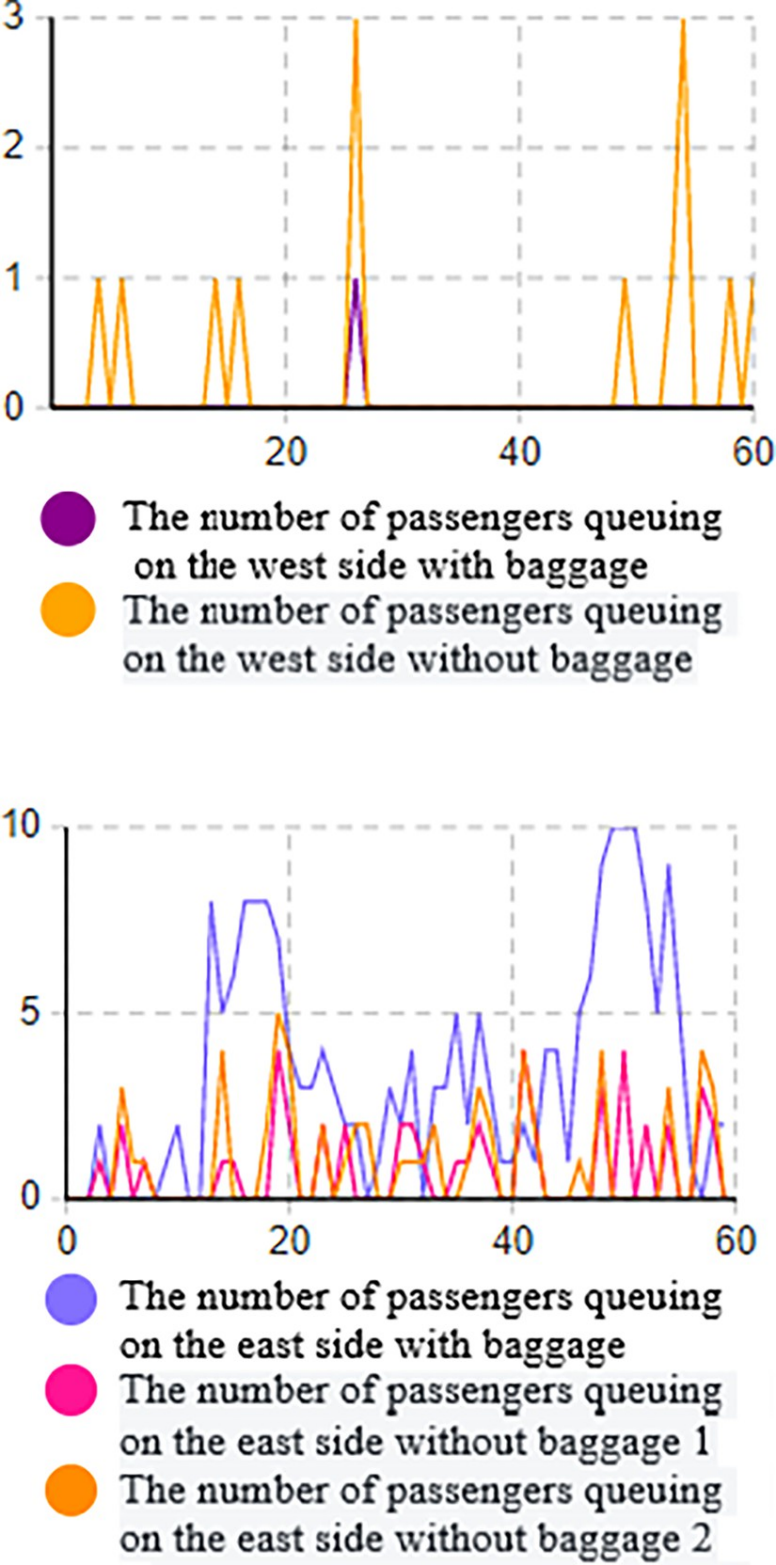
Simulation results of queueing of security check after optimization. (a)The number of passengers queuing for security check on the west side after optimization. (b) The number of passengers queuing for security check on the east side after optimization.

The line chart of the queue lengths for the optimized security check and ticket checking facilities (Figs 21 and 22) indicates that the queue length for the security check facilities on the west side generally ranges from 1 to 3 people, with a maximum queue length of 3 pe 3 people. On the east side, the queue length for the security check facilities typically ranges from 1 to 5 people, with a maximum queue length of 10 people. For the ticket checking facilities on the east side, the queue length generally ranges from 1 to 3 people, with a maximum queue length of 4 people, while on the west side, the queue length typically ranges from 1 to 2 people, with a maximum queue length of 2 people. Comparing these results with the output of the simulation experiment under the current situation, there has been a decrease in queue lengths for all facilities.

The dissipation time for the security check and ticket checking system on the west side has decreased to 30.54 minutes. On the east side, the dissipation time for the security check and ticket checking system has decreased to 30.87 minutes.

## 5 Results and discussion

Compare the optimized simulation results with the current simulation results based on the table below.

According to Table 7, it can be seen that the area density of security check facilities and ticket checking facilities before optimization is basically around 1.33 people/m^2^, while after optimization, it basically decreases to around 1.00 people/m^2^. The optimization level of each area is higher than 20%, indicating that after taking optimization measures, the passenger density in the security check and ticket checking areas during peak hours decreases, and congestion is alleviated.

**Table 7:** A comparison of thermal density index before and after optimization at Ciqunan Station.

evaluation indicator	Security check facilities on the west side	Ticket checking facilities on the west side	Security check facilities on the east side	Ticket checking facilities on the east side
Thermal density map before optimization (people/m^2^)	1.10	1.33	1.33	1.67
Thermal density map after optimization (people/m^2^)	0.67	1.00	1.00	1.33
Degree of optimization	39%	25%	25%	20%

According to Table 8, it can be observed that the longest queue length on the east side before optimization was 15 people, and on the west side, it was 7 people. After optimization, the maximum queue length on the east side was 10 people, and on the west side, it was 3 people, with optimization levels of 57% and 33% respectively. This indicates that during the morning peak hours, the queue lengths at the security check and ticket checking facilities have been reduced, leading to improved service efficiency and passenger entry efficiency.

**Table 8:** Comparison of the maximum queue length index before and after optimization at Ciqunan Station.

evaluation indicator	Security check facilities on the west side	Ticket checking facilities on the west side	Security check facilities on the east side	Ticket checking facilities on the east side
Maximum queue length before optimization/people	7	3	15	8
Maximum queue length after optimization/people/people	3	2	10	4
Degree of optimization	57%	33%	33%	50%

According to Table 9, it can be seen that for the peak period of passenger flow from 7:30 to 8:00, the dissipation time on the west side before optimization was 31.56 minutes, and on the east side, it was 36.12 minutes. After optimization, the dissipation time on the west side was reduced to 30.04 minutes, and on the east side, it was reduced to 30.87 minutes, with optimization levels of 3% and 15% respectively. This indicates that the queuing phenomenon formed by the passenger flow during the morning peak hours through the security check and ticket checking facilities system can dissipate quickly.

**Table 9:** Comparison of system dissipation time index before and after optimization at Ciqunan Station.

evaluation indicator	Security check facilities on the west side	Ticket checking facilities on the west side	Security check facilities on the east side	Ticket checking facilities on the east side
System dissipation time before optimization/min	31.56		36.12	
System dissipation time after optimization/min	30.54		30.87	
Degree of optimization	3%		15%	

The comparative analysis of the simulation results obtained from the current state simulation and optimization simulation shows that, with the implementation of optimization measures, the increase in the number of security check facilities results in an improvement in passenger security check efficiency. This leads to an increase in the number of passengers able to enter the station within a certain period of time and a decrease in the number of passengers lingering in bottleneck areas. As a result, the passenger flow density in the queue areas of the security check and ticket checking facilities on both the west and east sides decrease.

The addition of movable barriers can separate passengers with luggage from those without, effectively improving the security check efficiency for passengers without luggage. This reduces the delay time for passengers passing through the security check facilities, decreases the maximum queue length, overall enhancing passenger throughput efficiency, and also reduces the system dissipation time for passengers entering the station during peak periods. Therefore, optimization measures can effectively alleviate congestion during the morning peak hours, improve the service efficiency of security check and ticket checking facilities, and enhance passenger entry efficiency.

## 6 Conclusion and future works

### 6.1 Conclusion

Based on the investigation of pedestrian flow video data inside the metro station, this paper studied the distribution pattern of the time needed for passengers to pass through security check and ticket checking facilities, and then analyzed the characteristics of pedestrian flow at these facilities. A pedestrian flow model for rail transit stations was established based on the security check and ticket checking times, with passenger density at the facilities, maximum queue length, and system dissipation time selected as optimization evaluation indicators. Using AnyLogic simulation software, the morning peak-hour passenger flow process at the rail transit station was simulated, and the simulation parameters for the facilities and pedestrian flow were calibrated. Optimization measures for the case study station were proposed and simulated, and the feasibility of the optimization measures was verified through a comparison of the evaluation indicators.

### 6.2 Limitations and future works

This paper systematically studied the travel characteristics of fine-grained passenger behavior inside metro stations, and achieved certain theoretical results and application value. However, the passenger travel process within metro stations is a complex process, and factors such as the choice of queue lines and the varying sizes of carried luggage can lead to variations in the entering behaviors of different individual passengers. Based on the limitations of this study, further investigations can be conducted in the following aspects:

1. Strengthen towards passenger queuing theories. In real-life scenarios, passengers in the ticket checking area often switch queues temporarily due to the inability to complete ticket checking. Additionally, the proficiency of passengers with the ticket checking system can also affect the ticket checking time. Furthermore, there are instances of group travel among passengers in real-life scenarios, such as colleagues, couples, and children. These types of passenger behaviors differ from the travel characteristics of individual passengers. Further research can be conducted to explore the characteristics of these typical passenger behaviors.
2. The differentiation of passenger luggage attributes in this paper is based on whether the passenger’s luggage needs to undergo checking by X-ray machines. However, different sizes of luggage (backpacks, shoulder bags, suitcases) have varying degrees of impact on the time required for passengers to pass through security and ticket checking facilities. Future research can be conducted in this area.

## Supporting information

S1 File Original data obtained from the survey. (DOCX)
