# Supplementary material for: Towards simulation optimization of subway station considering refined passenger behaviors
Source: PLoS One. 2024 Jun 6;19(6):e0304081. doi: 10.1371/journal.pone.0304081 (PMC11156381; doi:10.1371/journal.pone.0304081)
Supplement: S1 File — (DOCX) [file pone.0304081.s001.docx]

Table 1 The passage time of pedestrians with different attributes at security check and ticketing area. (unit: second)

| Num. | Gender | | Num. | With or without bag | | Num. | Fare checking ways | |
| --- | --- | --- | --- | --- | --- | --- | --- | --- |
|  | M | F |  | With | Without |  | Card | Code |
| 1 | 3.10 | 3.11 | 1 | 2.65 | 3.80 | 1 | 2.86 | 2.97 |
| 2 | 3.15 | 3.12 | 2 | 2.67 | 3.24 | 2 | 2.93 | 3.02 |
| 3 | 3.28 | 3.03 | 3 | 2.66 | 3.29 | 3 | 2.90 | 2.99 |
| 4 | 2.80 | 2.91 | 4 | 2.76 | 3.20 | 4 | 2.83 | 3.28 |
| 5 | 2.87 | 2.81 | 5 | 2.28 | 3.26 | 5 | 2.84 | 3.25 |
| 6 | 2.75 | 2.89 | 6 | 2.36 | 3.30 | 6 | 2.88 | 3.02 |
| 7 | 2.86 | 3.51 | 7 | 2.29 | 3.10 | 7 | 2.85 | 3.00 |
| 8 | 2.92 | 2.53 | 8 | 2.38 | 3.76 | 8 | 2.87 | 3.03 |
| 9 | 2.61 | 3.90 | 9 | 2.42 | 3.72 | 9 | 2.91 | 3.39 |
| 10 | 2.73 | 2.57 | 10 | 2.46 | 3.60 | 10 | 2.93 | 3.37 |
| 11 | 2.94 | 2.63 | 11 | 2.32 | 3.57 | 11 | 2.78 | 3.21 |
| 12 | 2.37 | 2.31 | 12 | 2.31 | 4.07 | 12 | 2.80 | 3.25 |
| 13 | 2.76 | 2.31 | 13 | 2.34 | 3.83 | 13 | 3.04 | 3.24 |
| 14 | 2.75 | 3.38 | 14 | 2.35 | 3.85 | 14 | 3.06 | 3.49 |
| 15 | 2.83 | 2.75 | 15 | 2.37 | 8.93 | 15 | 3.07 | 3.31 |
| 16 | 2.33 | 2.25 | 16 | 2.32 | 8.18 | 16 | 2.76 | 3.24 |
| 17 | 2.60 | 2.47 | 17 | 2.38 | 8.04 | 17 | 2.80 | 3.54 |
| 18 | 2.43 | 2.31 | 18 | 2.34 | 9.04 | 18 | 2.89 | 3.56 |
| 19 | 2.29 | 2.55 | 19 | 2.36 | 7.85 | 19 | 2.91 | 3.63 |
| 20 | 2.18 | 3.13 | 20 | 2.35 | 9.48 | 20 | 2.87 | 3.65 |
| 21 | 2.34 | 2.89 | 21 | 2.66 | 10.63 | 21 | 2.93 | 3.55 |
| 22 | 2.47 | 2.97 | 22 | 2.68 | 10.70 | 22 | 3.06 | 3.57 |
| 23 | 2.14 | 3.00 | 23 | 2.68 | 10.27 | 23 | 3.08 | 3.66 |
| 24 | 2.23 | 3.11 | 24 | 2.76 | 9.90 | 24 | 3.24 | 3.68 |
| 25 | 2.48 | 3.13 | 25 | 2.86 | 10.25 | 25 | 3.21 | 3.64 |
| 26 | 3.11 | 3.09 | 26 | 2.68 | 8.44 | 26 | 2.83 | 3.63 |
| 27 | 3.29 | 2.87 | 27 | 2.76 | 10.25 | 27 | 2.86 | 3.65 |
| 28 | 4.02 | 2.92 | 28 | 2.78 | 9.06 | 28 | 3.19 | 3.61 |
| 29 | 2.68 | 2.94 | 29 | 3.06 | 10.31 | 29 | 3.21 | 3.24 |
| 30 | 2.75 | 3.02 | 30 | 3.09 | 9.45 | 30 | 3.10 | 3.20 |
| 31 | 2.15 | 3.04 | 31 | 3.02 | 8.50 | 31 | 3.13 | 3.33 |
| 32 | 2.54 | 2.96 | 32 | 3.07 | 8.59 | 32 | 3.28 | 3.39 |
| 33 | 2.27 | 2.94 | 33 | 3.16 | 11.16 | 33 | 3.27 | 3.27 |
| 34 | 2.41 | 3.16 | 34 | 2.02 | 11.62 | 34 | 3.23 | 3.02 |
| 35 | 2.24 | 3.26 | 35 | 2.07 | 8.38 | 35 | 3.27 | 3.04 |
| 36 | 2.35 | 3.50 | 36 | 2.06 | 11.87 | 36 | 3.21 | 3.47 |
| 37 | 2.11 | 3.05 | 37 | 2.17 | 8.39 | 37 | 3.12 | 3.45 |
| 38 | 2.26 | 3.15 | 38 | 2.09 | 8.34 | 38 | 3.15 | 3.45 |
| 39 | 3.41 | 3.16 | 39 | 1.73 | 8.04 | 39 | 3.13 | 3.42 |
| 40 | 3.00 | 3.12 | 40 | 1.92 | 9.03 | 40 | 3.16 | 3.83 |
| … | … | … | … | … | … | … | … | … |

Table 2. Collected passenger data (unit: person)

| With or without-bag | Gender | Number |
| --- | --- | --- |
| With bag | male | 50 |
|  | female | 41 |
| Without-bag | male | 237 |
|  | female | 200 |
| Fare checking ways | Gender | Number |
| Card | male | 232 |
|  | female | 168 |
| Code | male | 198 |
|  | female | 215 |
